# The effect of cystic echinococcosis (hydatid disease) on carcase weight in cattle in eastern Australia

**DOI:** 10.1038/s41598-024-57886-2

**Published:** 2024-03-27

**Authors:** Victoria J. Brookes, Tamsin S. Barnes, David J. Jenkins, Matthew R. Van der Saag, Robert Dempster, Cara S. Wilson

**Affiliations:** 1 Sydney School of Veterinary Science, Faculty of Science, The University of Sydney, Camperdown, NSW 2008 Australia; 2 The University of Queensland, School of Veterinary Science, Gatton, QLD 4343 Australia; 3 Epivet Pty. Ltd., Withcott, QLD 4352 Australia; 4 School of Agricultural, Environmental and Veterinary Sciences, Faculty of Science and Health, Charles Sturt University, Wagga Wagga, NSW 2678 Australia; 5 Meat and Livestock Australia, Level 1, 40 Mount Street, North Sydney, NSW 2060 Australia; 6 Virbac (Australia) Pty Ltd, 361 Horsley Road, Milperra, NSW 2214 Australia; 7 CQUniversity Australia, Institute for Future Farming Systems, Rockhampton, QLD 4702 Australia; 8 Sydney Infectious Diseases Institute, Faculty of Medicine and Health, University of Sydney, Westmead, NSW, 2145, Australia

**Keywords:** Parasite host response, Risk factors

## Abstract

Cystic echinococcosis is caused by the zoonotic tapeworm *Echinococcus granulosus.* There has been ongoing controversy over whether it causes weight loss in cattle. Recently implemented recording of comorbidities at processors has provided opportunity to investigate this effect. Using prevalence-based observational data from 1,648,049 adult cattle processed in seven states and territories in Australia (2019–2022), we explored associations between carcase weight, hydatid cysts, comorbidities, sex, age, and region. Linear mixed-effect regression models estimated the effect of cystic echinococcosis on carcase weight, guided by directed acyclic graphs to reduce bias. The highest, previously unreported, prevalence was in the southeast Queensland region. The estimated effect of cystic echinococcosis cysts on carcase weight ranged from a gain of 0.32 kg/carcase (standard error [se] 0.58 kg; two-tooth 2022) to a loss of −5.45 kg/carcase (se 0.63 kg; six-tooth 2019) with most point estimates (11/16) between 0 and −2.5 kg across all cattle grouped by year and dentition. This effect size would be practically undetectable in live cattle which is an important finding; cattle producers are unlikely to observe increased productivity through weight gain from cystic echinococcosis prevention in cattle, and awareness to strengthen prevention in domestic dogs around cattle properties to reduce human risk remains a public health focus.

## Introduction

*Echinococcus granulosus* is a tapeworm that causes the parasitic zoonosis, cystic echinococcosis. There are several species of *Echinococcus* globally, but only *Echinococcus granulosus* sensu stricto has been reported in Australia. Canid species are definitive hosts, harbouring the adult tapeworm. In Australia, these are domestic dogs, dingoes, their hybrids, and foxes. Herbivorous or omnivorous species are intermediate hosts in which the larval stage of the parasite develop within fluid-filled (hydatid) cysts in the viscera (offal). In Australia these are predominantly macropods, sheep, and cattle^1,2^. Although cattle are considered accidental hosts and infection is generally subclinical, hydatid cysts are frequently found in the viscera at processing^1,3^, where they are primarily reported in the liver and lungs^3,4^. Cysts are less frequently reported in the heart, kidney, spleen^1,3,5^ and occasionally the brain and skeletal muscle^1,6^.

Factors associated with cattle and their environment influence the probability of hydatid cysts at processing. For example, the prevalence of hydatid cysts at processing has been reported to be higher in older cattle and those that are grass-fed^3,4,7,8^. Reported prevalence in cattle < 1 year old is low (< 3%)^3,9^, but for those that are > 4 years, prevalence of up to 39.5% has been reported^4,8^, and a recent study reported that the true prevalence in eight-tooth animals (estimated > 3.5 years) could be as high as 85.6% (Wilson, et al.^3^). The prevalence of hydatid cysts at processing also varies with geographic origin of the cattle, with higher prevalence regions including the Great Dividing Range and along the northern coast of New South Wales^1,3,4,9^. This has been attributed to the distribution of wild dogs, macropods (more abundant in regions encroaching on the hills of the Great Dividing Range), and more favourable climatic conditions for survival of the environmental stage of the parasite^4,9^. Overall, previous estimates of prevalence have generally been lower than more recent estimates which have accounted for the sensitivity and specificity of meat inspection^1,3,4,7^.

Understanding the impact that cystic echinococcosis has on the weight of cattle has been constrained by the use of observational data and study design, and often, evidence to support claims that cystic echinococcosis causes weight loss has been weak or non-existent. Although it was reported that cystic echinococcosis reduced productivity in cattle in a case-study in Cyprus, the source population from which this was inferred was not described^10^. Similarly, in a review of the economic impact of hydatid infection in production animals, a reduction in carcass weight was also reported (up to 20%); however, supporting studies were not available^11^. A study conducted in 2006 estimated that globally, annual economic losses from cystic echinococcosis in livestock could be greater than USD 2 million due to liver condemnation, decreased carcase weight, decreased fecundity, reduced milk production and decreased hide value^12^, although there was great uncertainty about the effect of cystic echinococcosis on some parameters, including carcase weight. Whilst the effects of productivity losses due to offal condemnation can be directly measured, an effect of cystic echinococcosis on carcase weight has been difficult to estimate because other causes of reduced carcase weight such as concurrent disease, sex, system (grass fed *versus* feedlot), and cattle origin have not been recorded^13^. For example, unadjusted estimates of up to 16.2 kg reduction in carcase weight in cattle with cystic echinococcosis have been reported in Australian studies^14,15^. Recently however, multi-condition reporting for carcase and offal defects has been instigated in some Australian processors, whereas previously, processors could only report the main reason for downgrading or condemnation of carcases and offal at processing and would most likely record the predominant lesion. With the inclusion of other key variables that affect cattle carcase weight (for example, sex, location and system, breed and age^16^), this provides an opportunity to more accurately estimate the impact of cystic echinococcosis on weight using observational data. Understanding the impact that cystic echinococcosis has on the weight, and therefore, the productivity of cattle is important for prevention and control, because it influences adoption of control strategies, such as deworming of domestic dogs, on-farm wild dog control, and potentially, the vaccination of cattle against infection with *Echinococcus*^17,18^.

To determine the impact of cystic echinococcosis on the weight of cattle, it would be ideal to conduct a randomised controlled trial in which the weight of some cattle that were randomly infected with *E. granulosus* could be compared with others that were not, (such that the only difference between the groups was cystic echinococcosis). However, this has low feasibility due to the long duration of this disease. Therefore, observational data such as abattoir records provide the next best option for causal inference. However, it is well known that observational data are prone to bias—infection with *E. granulosus* is not random so confounding occurs due to other causes of weight loss that are also associated with cystic echinococcosis, the analysed cattle might not be a representative sample of the source population (selection bias), and parameters might be measured or classified incorrectly (information bias). Recently, the use of directed acyclic graphs (DAGs) has become more prominent to avoid, or at least inform, sources of bias in observational studies through directing correctly structured analyses^19^. A recent study highlighted the challenges of statistical analysis without causal thinking and demonstrated how the understanding underlying structure of data (for example, using DAGs) is critical for causal inference^20^.

The objective of this study was to estimate the effect of the presence of hydatid cysts on carcase weight of cattle processed at several processors in eastern Australia using observational data with analyses guided by a DAG. These processors were selected because they report multiple comorbidities in carcases and the viscera, not only the predominant cause of downgrading or condemnation. This study has implications for public health as well as cattle productivity; if cystic echinococcosis reduces the carcase weight of cattle, producers will have more incentive to prevent disease in their cattle by controlling *Echinococcus* in dogs, which also reduces the risk of infection of people.

## Methods

We hypothesised that the presence of hydatid cysts (infection with *Echinococcus granulosus* sensu stricto) in beef cattle reduces carcase weight at processing. The exposure variable of interest was the presence of hydatid cysts (measured as hydatid cysts reported at processing), and the outcome of interest was hot standard carcase weight (HSCW: weight within two hours of slaughter following standard trim) at processing. The target population was beef cattle in Australia, and the source population was adult beef cattle processed between 2 January 2019 and 26 July 2022 at five abattoirs in eastern Australia that had remained in the same region for their lifetime.

Eligible cattle had the same property identification code (PIC) region recorded at birth and prior to processing; we made the assumption that these cattle were likely to have stayed in that PIC region for their lifetime. PIC codes are unique property identifiers for properties on which livestock are held, incorporating spatial identifiers representing regions in which the property is located (here, referred to as ‘PIC region’). As part of Australia’s National Livestock Identification System (NLIS), all cattle are individually identifiable by an electronic device (generally an ear tag), all physical locations on which livestock are held are identified by means of a PIC, and all livestock location data and movements are recorded in a central database^21,22^.

Ethical approval was not required for this study. Data were obtained for cattle that were processed in the beef supply chain for human consumption not for the purposes of this study, and no samples were collected from cattle.

### Data collection and cleaning

Data were provided for 3,364,737 cattle processed between 2 January 2019 to 26 July 2022 at five processors (three sites in Queensland, one site in New South Wales, and one site in South Australia). Following data cleaning in Excel^23^ including checking for data consistency and removal of duplicates, cattle that had the same PIC region recorded at birth and prior to processing were selected.

Cattle were removed from the dataset if they were likely to be vealers (not weaned for more than seven days and no evidence of eruption of permanent incisor teeth; https://www.mla.com.au/general/glossary/#glossarySection_V, accessed 1 June 2023) by removing cattle with zero-tooth and HSCW < 150 kg, or any cattle with HSCW < 50 kg (likely vealers but dentition misclassified). A subset of 1,648,049 cattle for analysis remained.

Variables recorded for each animal relevant to the study were PIC at birth and prior to processing, processor, type of animal (vealer or beef), sex, dentition (zero-, two-, four-, six-, and eight-tooth), whether the animal was grain or grass fed, the presence of cystic echinococcosis and organs infected, the presence of other pathological conditions and causes of downgrading and condemnation including liver fluke (comorbidities), and HSCW. The presence of hydatid cysts was categorised to a single binary variable according to whether hydatid cysts were detected in any organ, following assessment of the proportion of cattle with hydatid cysts in the liver relative to other organs. Conditions other than fluke that were identified at processing and could have influenced carcase weight (comorbidities) were categorised as a binary variable for individual cattle according to whether there was presence of at least one comorbidity. We made no assumption about the severity of comorbidities other than a reduced carcase weight would be expected either due to smaller size (reduced growth) or trimming. Comorbidities included arthritis, bruising, and other carcass defects such as cancer, anaemia, antibiotic treatment, fracture, and myositis (see Supplementary Material for the full list of comorbidities). Age was classified according to dentition with zero-, two-, four-, six-, and eight-tooth cattle of approximate ages < 18 months, 18–30 months, 24–36 months, 30–42 months, and ≥ 42 months, respectively^24^.

### Descriptive analyses

Descriptive statistical analyses of cattle characteristics, disease, and HSCW (outcome variable of interest) were conducted. Choropleth maps of characteristics of the cattle at processing from each PIC region were produced (QGIS v 3.28.2, https://qgis.org/en/site/). These were: the number of cattle, proportion of cattle in which hydatid cysts were detected (exposure variable of interest), proportion of cattle detected with fluke, proportion of cattle detected with comorbidities, proportion of grain-fed cattle, distribution of sex, dentition, and HSCW. Choropleth maps were also used to display the mode frequency of processor for cattle from each PIC region, and the Euclidean distance from PIC region centroid to processing site of cattle was calculated. A Kruskal–Wallis test (P > 0.05) was used to determine if the distribution of the Euclidian distances travelled by cattle from PICs to processors travelled differed between sites; otherwise (and in accordance with statistical rationale^25–27^), we avoid the use of statistical hypothesis testing in the descriptive analyses because the purpose of this section is to demonstrate patterns in covariates before adjustment for bias in the ‘statistical analyses’ section.

### Statistical analyses

All statistical analyses were conducted in R^28^ and packages tidyverse^29^, plyr^30^, ggplot^31^, lubridate^32^, epiR^33^, lme4^34^, Performance^35^, nlme^36^, and reshape2^37^. In this section, analyses were conducted with the aim of minimising bias to determine the causal effect of cystic echinococcosis on cattle carcase weight.

A directed acyclic graph (DAG; Figure S1) of variables that influence, or could be influenced by, both the identification of cystic echinococcosis at processing and HSCW was developed using previously published information about risk factors for cystic echinococcosis in cattle in Australia and consultation with cystic echinococcosis and beef cattle industry experts (DJ, MVdS, RD, JL, CW). The DAG was used to identify the minimal sufficient adjustment set of variables to estimate the total effect of the presence of hydatid cysts on HSCW using linear, mixed-effects regression models that accounted for potential sources of bias using the lmer function in the lme4 package in R^34^. Due to the way in which parameters are estimated (maximum likelihood) estimates and the unbalanced nature of the observational data in this study, P values were not calculated; standard errors of estimates and 95% confidence intervals are presented in tables and plots, respectively.

Initially, models were constructed using the entire dataset to broadly investigate the total effect of cystic echinococcosis on carcase weight with and without adjustment for confounding pathways, and to investigate the effect of clustering by PIC region. Model fit was assessed on the full model (all covariates included) using the entire dataset to evaluate assumptions about normality of residuals, normality of random effects, linear relationships, homogeneity of variance, and multicollinearity. Following this preliminary investigation, models to investigate the total effect of cystic echinococcosis on carcase weight which accounted for confounding pathways and clustering (by including PIC Region as a random effect) were constructed for groups of cattle of the same age (dentition) and year of processing so that the population in each group could be considered stable regarding exposure experiences (including covariates) and subsequent cystic echinococcosis incidence and duration. Plots of point estimates with 95% confidence intervals of the total estimated effect of cystic echinococcosis (detected at processing) on hot standard carcass weight and model summaries were reported.

## Results

### Processors

**Figure 1:**
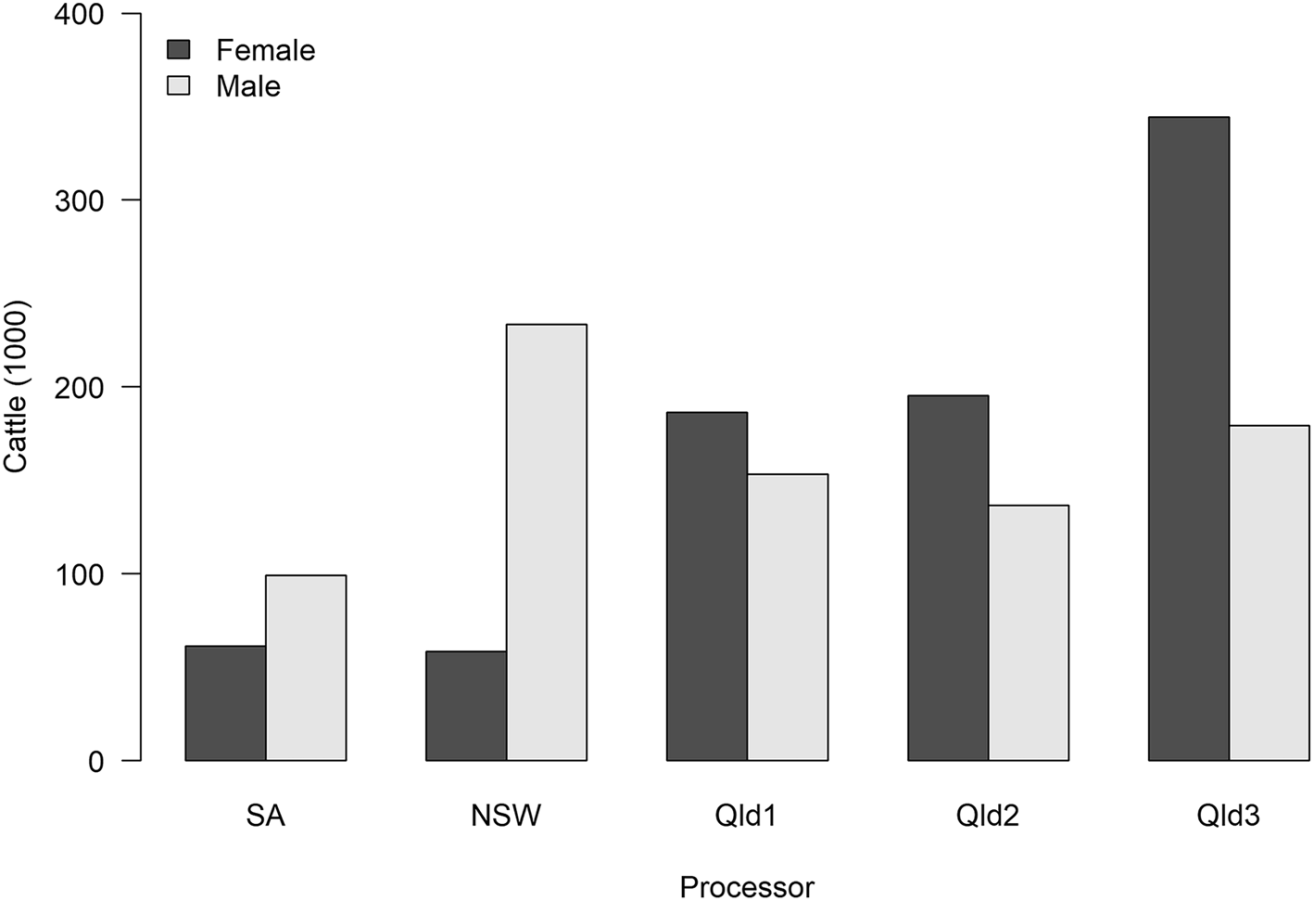
Number of adult cattle with the same property identification code (PIC) region recorded at birth and prior to processing, at each processor and stratified by sex, in a study of the effect of cystic echinococcosis on carcase weight at five processors in eastern Australia, 2019–2022.

Of the 1,648,049 eligible cattle, most were processed at the three Queensland sites (Qld1, Qld2, Qld3; n = 1,195,472, 72.5%; Fig. 1), of which the Qld3 site processed most (n = 523,772). The South Australian site processed the fewest cattle (n = 160,661). Most cattle were sourced from eastern regional and mid to mid-north coast Queensland (Figure S2). The median number of individual PICs in each PIC region was 49 (range 1–861), and the median number of cattle processed from each individual PIC was 1268 (range 1–218,627). The annual number of cattle processed at all the sites in the study decreased from 607,939 in 2019 to 196,263 in 2022 (2022 only includes data to 26 June 2022; Figure S3). The geographic extent of the source distribution for cattle processed at each site varied (Figure S4), and the median Euclidean distance of PIC region centroids from the processing site varied significantly between sites (Kruskal Wallis rank sum test Χ^2^ 194,994, df 4, P < 0.01; Figure S5).

### Cattle characteristics

**Figure 2:**
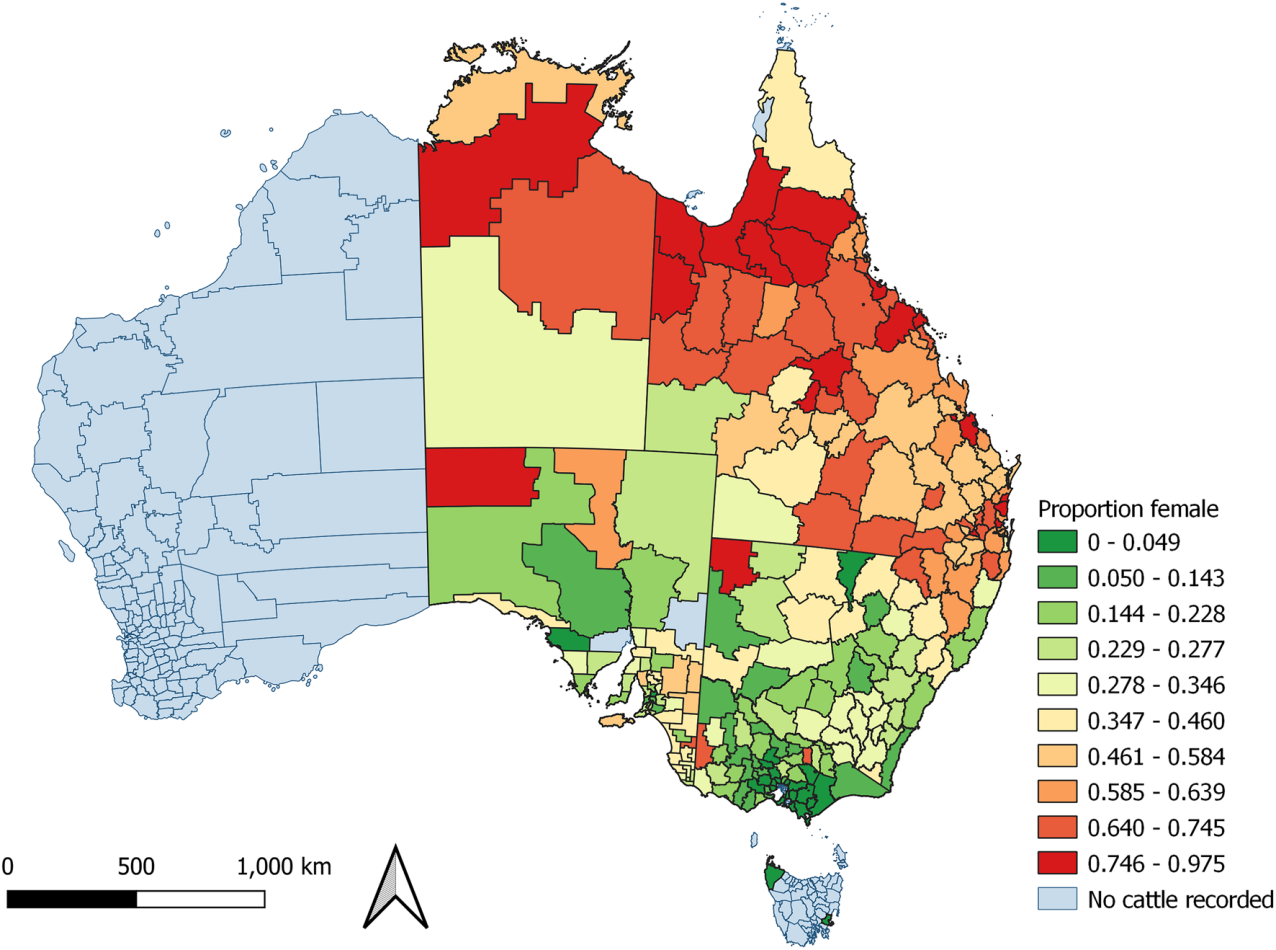
Proportion of female cattle with the same property identification code (PIC) region recorded at birth and prior to processing, by PIC region, in a study of the effect of cystic echinococcosis on carcase weight at five processors in eastern Australia, 2019–2022. Map made by authors using QGIS^50^.

**Figure 3:**
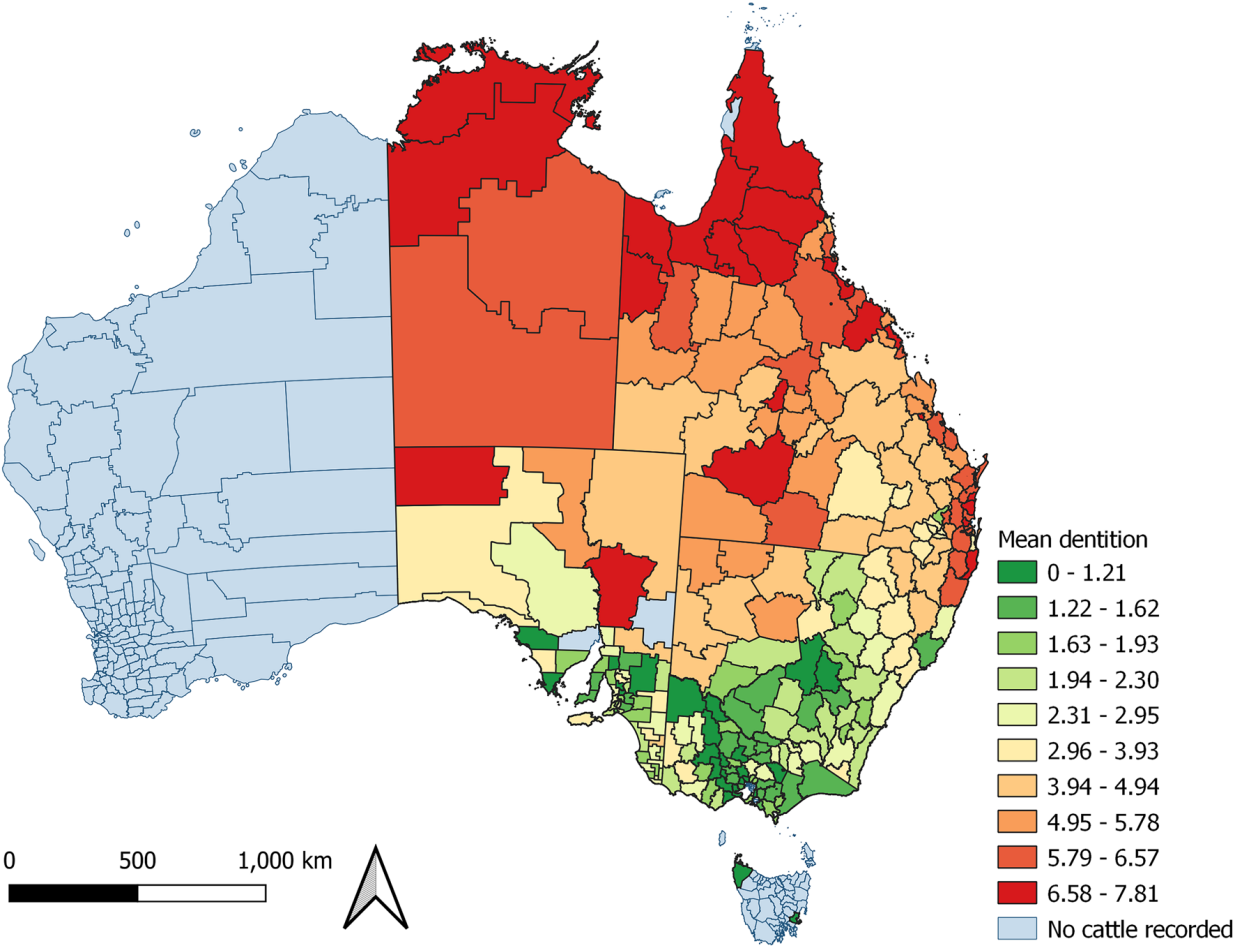
Age of cattle by mean dentition (one–eight teeth) with the same property identification code (PIC) region recorded at birth and prior to processing, by PIC region, in a study of the effect of cystic echinococcosis on carcase weight at five processors in eastern Australia, 2019–2022. Map made by authors using QGIS^50^.

**Figure 4:**
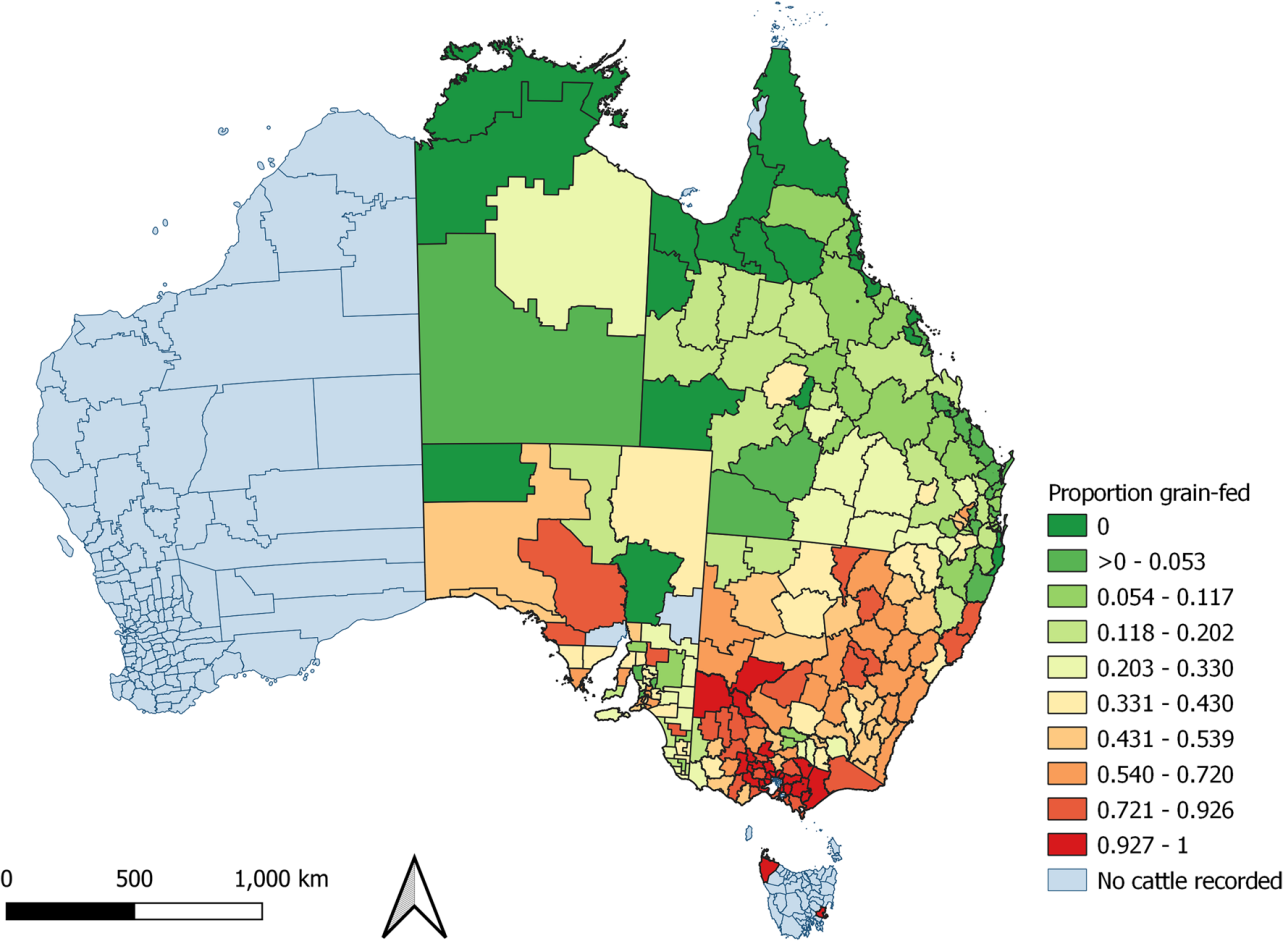
Proportion of grain-fed cattle with the same property identification code (PIC) region recorded at birth and prior to processing, by PIC region, in a study of the effect of cystic echinococcosis on carcase weight at five processors in eastern Australia, 2019–2022. Map made by authors using QGIS^50^.

Figure 2 shows the proportion of cattle by sex from each PIC region. A greater proportion of cattle were female from PIC regions in Queensland and the Northern Territory than from southern states. This is reflected in the proportion of female cattle processed at each site; most cattle processed at Queensland sites were female (n = 726,215, 60.7%; Fig. 1), and most cattle processed at the New South Wales and South Australia sites were male (n = 332,675, 73.5%) (Fig. 1). By dentition and sex, the largest group of cattle were eight-tooth females (n = 479,011), and the smallest was eight-tooth males (n = 60,757; Figure S6). Figure 3 shows that older cattle were more likely to have been sourced from northern PICs. The proportion of cattle that had been grain-fed also varied by region, with grain-fed cattle commonly being from southern PIC regions (Fig. 4).

**Figure 5:**
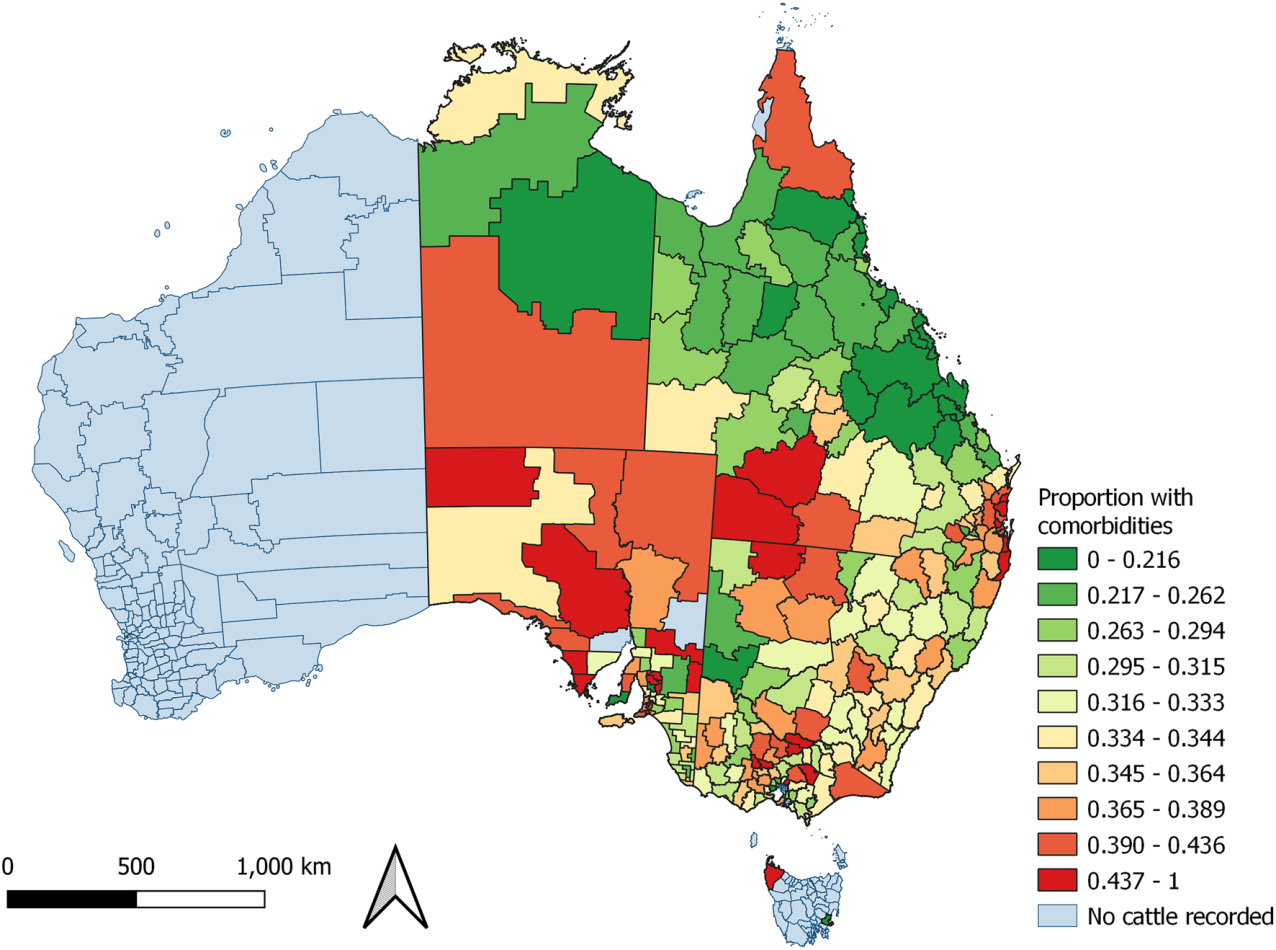
Proportion of cattle with the same property identification code (PIC) region recorded at birth and prior to processing with comorbidities (disease conditions other than hydatid cysts or liver fluke) detected at processing from each PIC region, in a study of the effect of cystic echinococcosis on carcase weight at five processors in eastern Australia, 2019–2022. Map made by authors using QGIS^50^.

### Disease detection

**Figure 6:**
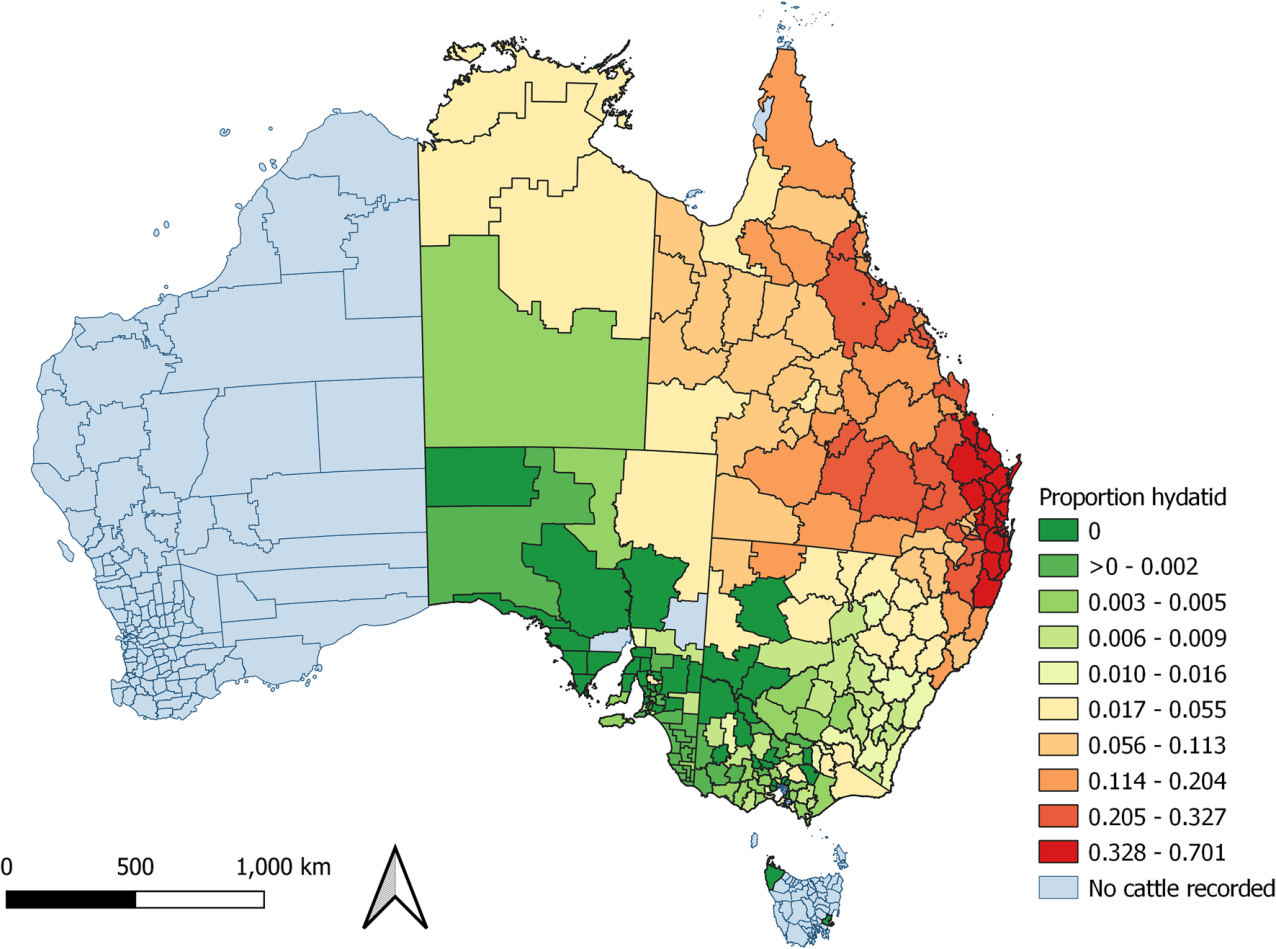
Proportion of cattle with the same property identification code (PIC) region recorded at birth and prior to processing with hydatid cysts detected at processing from each PIC region, in a study of the effect of cystic echinococcosis on carcase weight at five processors in eastern Australia, 2019–2022. Map made by authors using QGIS^50^.

**Figure 7:**
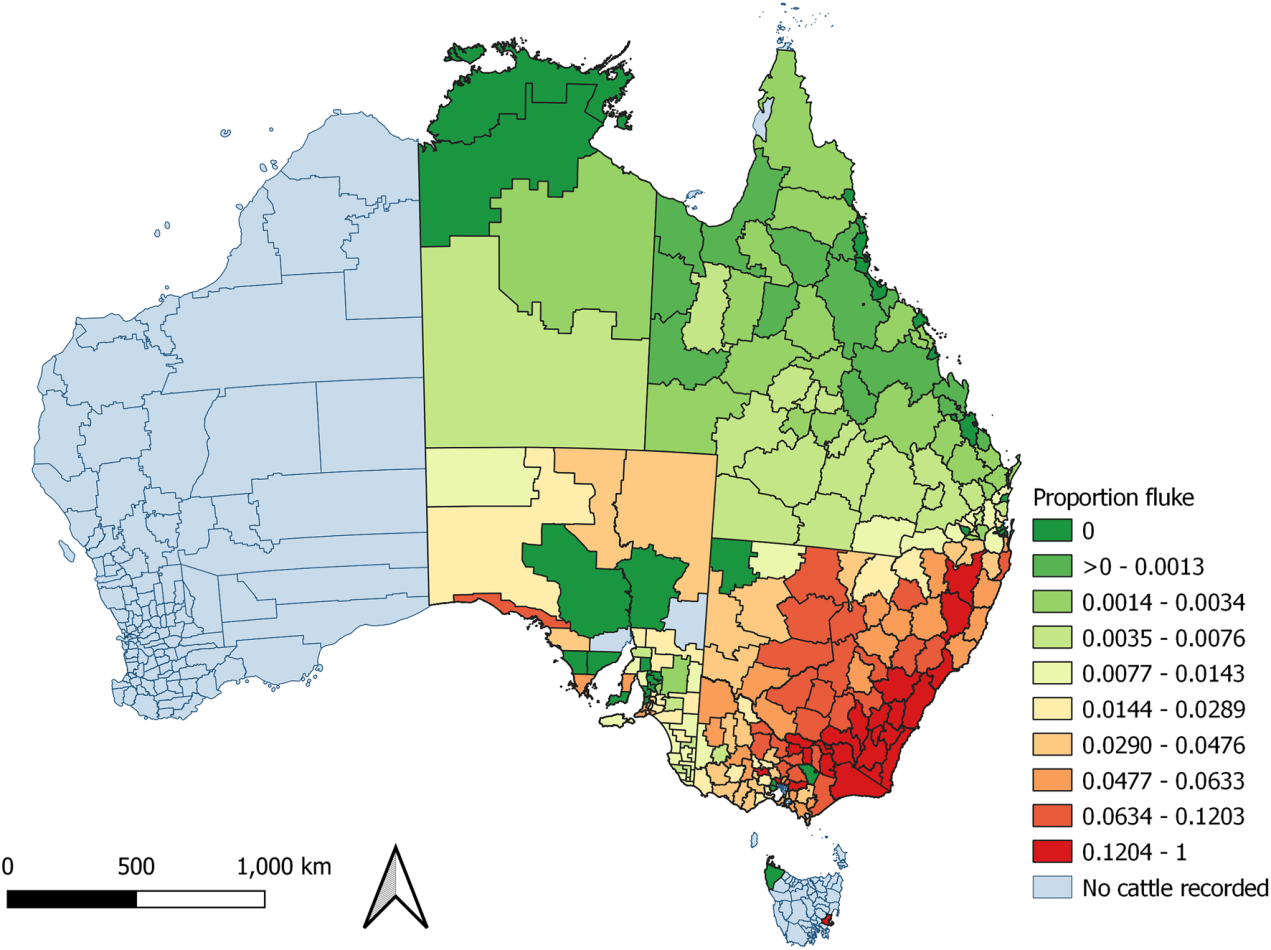
Proportion of cattle with the same property identification code (PIC) region recorded at birth and prior to processing with liver fluke detected at slaughter from each PIC region, in a study of the effect of cystic echinococcosis on carcase weight at five processors in eastern Australia, 2019–2022. Map made by authors using QGIS^50^.

The proportion of cattle with comorbidities detected at processing varied by PIC region (Fig. 5). In contrast, the proportion of cattle detected with hydatid cysts and liver fluke demonstrated a strong spatial pattern (Figs. 6 and 7). Hydatid cysts were more commonly detected in cattle from northern NSW and southeast and coastal Queensland, and carcases with liver fluke were more commonly detected in New South Wales and Victoria, especially southwest coastal regions. Of particular interest was the high proportion of cattle with hydatid cysts detected in the Brisbane region (Figure S7). Although fewer cattle were processed from this region, the proportion of cattle in which hydatid cysts were detected was consistently high (33–70%). The proportion of all cattle with hydatid cysts detected in any organ was 17.2% (n = 283,073). Of these, 94% of cattle had cysts detected in the liver (44% in liver and lung), and 6% had cysts detected in the lung only (S8). A negligible number of cattle had cysts detected in the spleen (n = 44), or heart (n = 29), and of these, most also had hydatid detected in the liver (n = 67; 92%). The proportion of cattle detected with hydatid cysts increased with age (S9). Hydatid cysts were also more commonly detected in cattle that had not been grain-fed, and in female cattle (Figure S10). Female cattle were less commonly grain-fed (Figure S10) and a higher proportion of them were older (eight-tooth) cattle (Figure S6).

### Carcase weight

**Figure 8:**
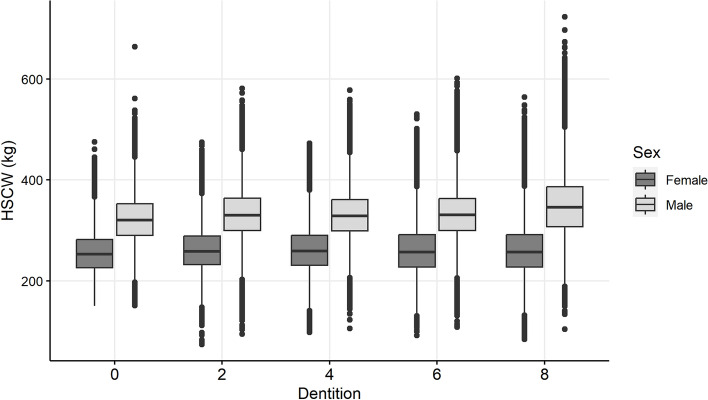
Carcase weight (HSCW = hot standard carcase weight) of cattle with the same property identification code (PIC) region recorded at birth and prior to processing, stratified by sex, in a study of the effect of cystic echinococcosis on carcase weight at five processors in eastern Australia, 2019–2022.

**Figure 9:**
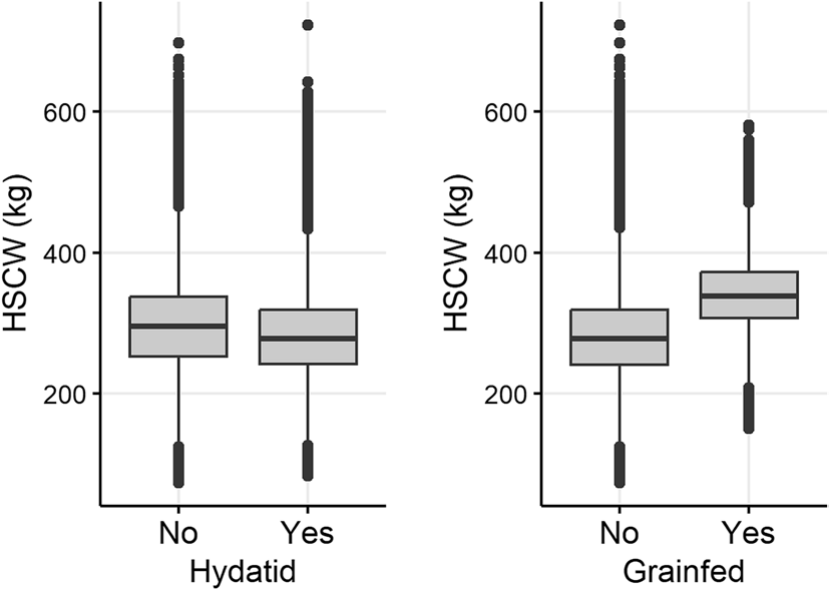
Carcase weight (HSCW = hot standard carcase weight) of cattle with the same property identification code (PIC) region recorded at birth and prior to processing, by the presence of hydatid cysts at processing (left) and whether they were grain-fed (right), in a study of the effect of cystic echinococcosis on carcase weight at five processors in eastern Australia, 2019–2022.

Hot standard carcase weight (HSCW) was similar between all age groups (Fig. 8). Female cattle were generally lighter, with mean HSCW 261.29 kg (95% range 182.4–363.6 kg, n = 846,117 cattle) compared to male cattle with a mean HSCW of 330.19 kg (95% range 235.8–433.4 kg, n = 801,932 cattle). Carcases from cattle that were not grain-fed were also lighter than grain-fed cattle (Fig. 9). Mean HSCW in grain-fed cattle was 340.4 kg (95% range 245.8–439.4 kg; n = 353,211 cattle), and mean HSCW in non-grain-fed cattle was 282.4 kg (95% range 188–379.9 kg, n = 1,294,828). Carcase weight was generally lower in carcases in which hydatid cysts were detected by approximately 14 kg (Fig. 9). Mean HSCW in cattle in which hydatid cysts were detected was 283.0 kg (95% range 189.2–404 kg; n = 283,073 cattle), and mean HSCW in cattle in which hydatid cysts were not detected was 297.3 kg (95% range 192–417.5 kg, n = 1,364,976).

**Figure 10:**
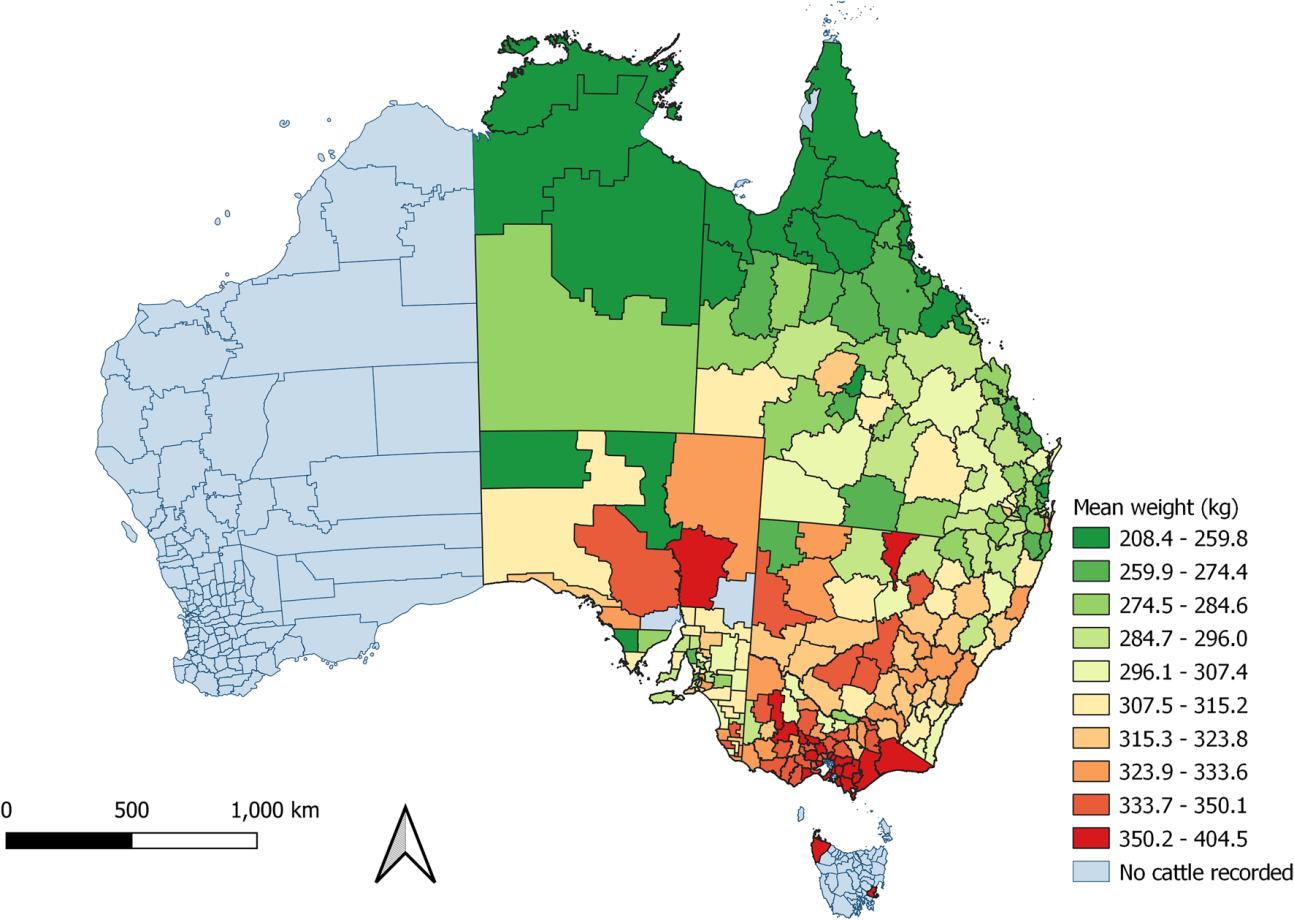
Mean hot standard carcase weight of cattle which had remained on the same property identification code (PIC) region for their lifetime, from each PIC region in a study of the effect of cystic echinococcosis on carcase weight at five processors in eastern Australia, 2019–2022. Map made by authors using QGIS^50^.

There was a visual association between the monthly proportion of female cattle, cattle that were not grain-fed, the mean age of cattle, the mean carcase weight of cattle and the proportion of cattle in which hydatid cysts were detected (Figure S11). These findings are consistent with the strong spatial pattern of the mean weight of cattle processed increasing from northern to southern PICs (Fig. 10), where cattle in the northern PICs were more likely to be female, older, and not grain-fed (Figs. 2, 3 and 4). This is also the region in which hydatid cysts were more commonly detected in processed cattle (Fig. 6).

### Statistical analyses

#### Preliminary statistical models

Models to explore clustering and the unadjusted (confounding not considered) association of cystic echinococcosis on HSCW were conducted on the entire dataset of 1,648,049 adult beef cattle that had the same PIC region recorded at birth and prior to processing (Table 1).

**Table 1:** Results from preliminary linear regression models of cattle which had the same property identification code (PIC) region recorded at birth and prior to processing, in a study of cattle processed at five processors in eastern and southern Australia in 2019.

Model	(A) Univariable	(B) Full	(C) Null mixed	(D) Full mixed
	Fixed effect: hydatid detection; no random effect	Fixed effect: hydatid detection + all covariates; no random effect	No fixed effects; random effect: PIC region	Fixed effects: hydatid detection + all covariates; random effect: PIC region
HSCW, kg	Estimate (se)	Estimate (se)	Estimate (se)	Estimate (se)
Intercept	297.26 (0.05)	− 18146.12 (66.72)	303.26 (2.51)	− 18146.12 (66.72)
Hydatid detected	− 14.25 (0.12)	− 4.46 (0.10)	–	− 4.46 (0.10)
Covariates: sex, age (dentition), year, presence of comorbidities, presence of fluke, abattoir, grain-fed (yes/no)				
Random effects				
σ2	–	–	2949.25	1793.78
τ00	–	–	1308.18	392.85
ICC	–	–	0.31	0.18
N	–	–	211	211
Observations	1,648,048—all models			
Marginal R2/conditional R2	0.000/0.307	0.008/0.008	0.002/0.302	0.380/0.491

HSCW, hot standard carcase weight; se, standard error; σ2, random effect variance; τ00, random intercept variance; ICC, intra-class correlation coefficient; N, number of random effect groups; R2, r-squared value.

The univariable, fixed effect, linear regression model indicated that without adjustment for confounding pathways, the association of the identification of cystic echinococcosis on carcase weight appeared to be large (carcases in which hydatid cysts were detected were on average 14.25 kg [standard error, se 0.12 kg] lighter than carcases in which hydatid cysts were not detected; Table 1, A), consistent with univariable descriptive analyses (Fig. 9). However, in the full model (Table 1, B), which included year of processing and covariates to reduce confounding as fixed effects (identified in the DAG [Figure S1]: sex, presence of comorbidities, presence of fluke, abattoir, grain-fed or not, PIC region), the total effect estimate of cystic echinococcosis on carcase weight was − 4.46 kg (carcases in which hydatid cysts were detected were on average 4.46 kg [se 0.1 kg] lighter than carcases in which hydatid cysts were not detected).

Models in which PIC region was included as a random effect indicated that carcase weight was moderately clustered by PIC region: the intraclass correlation coefficient (ICC) was high in the null model (Table 1; C) relative to the full model (Table 1; D), at 0.31 and 0.18, respectively. This is also consistent with the descriptive analyses – carcase weight was strongly associated with PIC region (Fig. 10), as were covariates grain-fed, sex, hydatid, and fluke detection (Figs. 2, 3, 4, 6, 7). Model assumptions held for normality of residuals, normality of random effects, linear relationship, homogeneity of variance, and lack of multicollinearity (Figure S12).

#### Final statistical models

Due to the nature of the data (prevalence rather than incidence of hydatid cysts), models with subgroups of cattle by age (dentition) and year were constructed to estimate the total effect of hydatid cysts on hot standard carcase weight by age and annual cohort. All models included covariates (sex, presence of comorbidities, presence of fluke, abattoir, grain-fed or not) to adjust for confounding pathways, and a random effect of PIC region.

**Figure 11:**
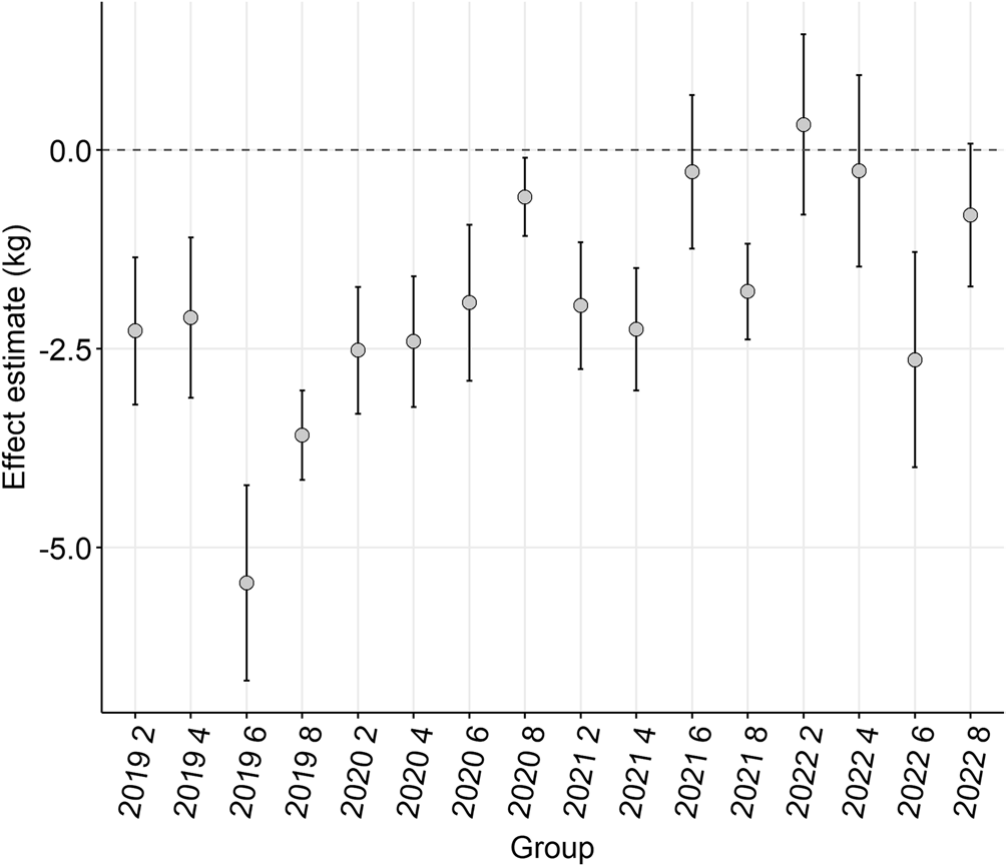
Point estimates and 95% confidence intervals of total effects of the presence of hydatid cysts (detected at processing) on hot standard carcase weight (HSCW; kg) in linear mixed-effects regression analyses of cattle stratified by age (dentition) and year, which had the same property identification code (PIC) region recoded at birth and prior to processing. Models are listed by year (2019–2022) and age (dentition; two-eight tooth). Models included fixed effects covariates: sex, presence of comorbidities, presence of fluke, abattoir, grain-fed (yes/no), and random effect PIC region.

The point estimates of the total effect of the presence of hydatid cysts ranged from − 5.45 kg (se 0.63 kg) to 0.32 kg (se 0.58 kg), in six-tooth cattle in 2019 and two-tooth cattle in 2022, respectively. Most point estimates (11 of 16) were between − 2.5 and 0 kg (Fig. 11). Model summaries (Tables S1–S4) indicate clustering by PIC region, with ICCs ranging from 0.24 – 0.38. Plots of fixed effect coefficients for all covariates in the models are included in Supplementary Material for complete information about each model but should not be interpreted as effect estimates for these covariates (Figures S13–S16).

## Discussion

The geographic extent of cattle sourced in this dataset and the recording of comorbidities including fluke as well as hydatid cysts during processing provided greater opportunity to investigate cystic echinococcosis than has previously been possible. We estimated that in each age group and year of processing, carcase weight was lighter by approximately 0 – 2.5 kg in cattle in which hydatid cysts were detected. It has been assumed that infections in which there are many and large cysts (which are more likely in older cattle due to the duration over which cysts have developed) will have a greater effect on weight, if there is any effect at all^38^. However, it is also possible that earlier, active infections in younger cattle stimulate an immune response to the disease process (and thus, cause reduced weight, as can be observed with other diseases in cattle^39,40^) than chronic infections in older cattle in which cysts are already walled off. The level of infection was not differentiated in cattle in the current study although was explored in a previous study in cattle processed in Australia from a wide geographic catchment area^38^. It was reported that cattle typically have light infections (few and small cysts), but it is the heavier infections that are more likely to be reported by the meat inspector regardless of age group^38,41^. However, whilst the estimated effect was consistent across age groups throughout the study years and is biologically plausible, the finding is based on observational data in which prevalence of cystic echinococcosis, not incidence, was reported. Therefore, we discuss this limitation and potential sources of bias below.

Another new finding from this study was that the highest apparent prevalence of hydatid cysts at processing centered on cattle sourced from the Brisbane region. Whilst high prevalence has been demonstrated previously along the Great Dividing Range and coastal northern New South Wales and most of the Queensland coastline, as well as regional Queensland, previous studies have not focused or have had few cattle from the Brisbane region^3,4^. Our study provides more comprehensive evidence that the distribution of cystic echinococcosis in cattle is consistent with the distribution of wild dogs and dingoes along the Great Dividing Range and coast where the climate is conducive to survival of *Echinococcus* eggs on pasture.

Residual confounding of the estimated total effect of hydatid cysts on carcase weight in this study is possible despite accounting for major variables that influence both carcase weight and the probability of infection with hydatid cysts (sex, grass or grain-fed, and comorbidities including fluke) in the statistical models. For example, breed of cattle was unknown, and is associated with geographic locality^4^; *Bos indicus* breeds of cattle are more often reared in northern Australia where cystic echinococcosis is more prevalent, and could be lighter by age than *Bos taurus* breeds in southern Australia. Factors such as breed can be considered as management decisions that are influenced by the farm location (producers rear breeds suited to the regional climate, topography, and vegetation) and therefore, were broadly accounted for by the inclusion of PIC region in the statistical model. However, variation within PIC regions will occur. For example, cattle that are less well grown for age could also be more likely to come from areas where they are more likely to become infected with hydatid cysts, such as marginal grazing areas where hosts such as wild dogs and macropods or sheep are present. Data about proximity to unimproved land on which wild dogs could roam, types of fencing that might allow dogs to access cattle properties, and the presence of domestic dogs on the property were not available. Therefore, whilst farms within PIC regions are broadly similar, heterogeneity within PIC regions could explain the apparent lower weight of carcases from cattle with cystic echinococcosis.

We also considered the possibility of selection bias of the estimated effect of cystic echinococcosis on carcase weight through a variety of mechanisms. If cattle die on farm, either due to disease or home slaughter, this could be considered as ‘loss to follow-up’ in the study population, and an additional association with the probability of this loss to follow-up with exposure to hydatid cysts would result in a biased estimate of the effect of cystic echinococcosis on carcase weight. However, given the insidious nature of cystic echinococcosis (inapparent clinical signs), the probability of cattle dying due to cystic echinococcosis is negligible. In contrast, home slaughter could have an association with cystic echinococcosis if it is more likely practiced by non-commercial producers in marginal cattle-rearing areas where cattle will also be lighter due to poorer grazing as well as also more likely to be infected with hydatid cysts. However, given the small population of home-slaughtered cattle relative to the number that are processed commercially in Australia, we believe that bias of the estimated effect of hydatid cysts on carcase weight due to this would be negligible.

**Figure 12:**
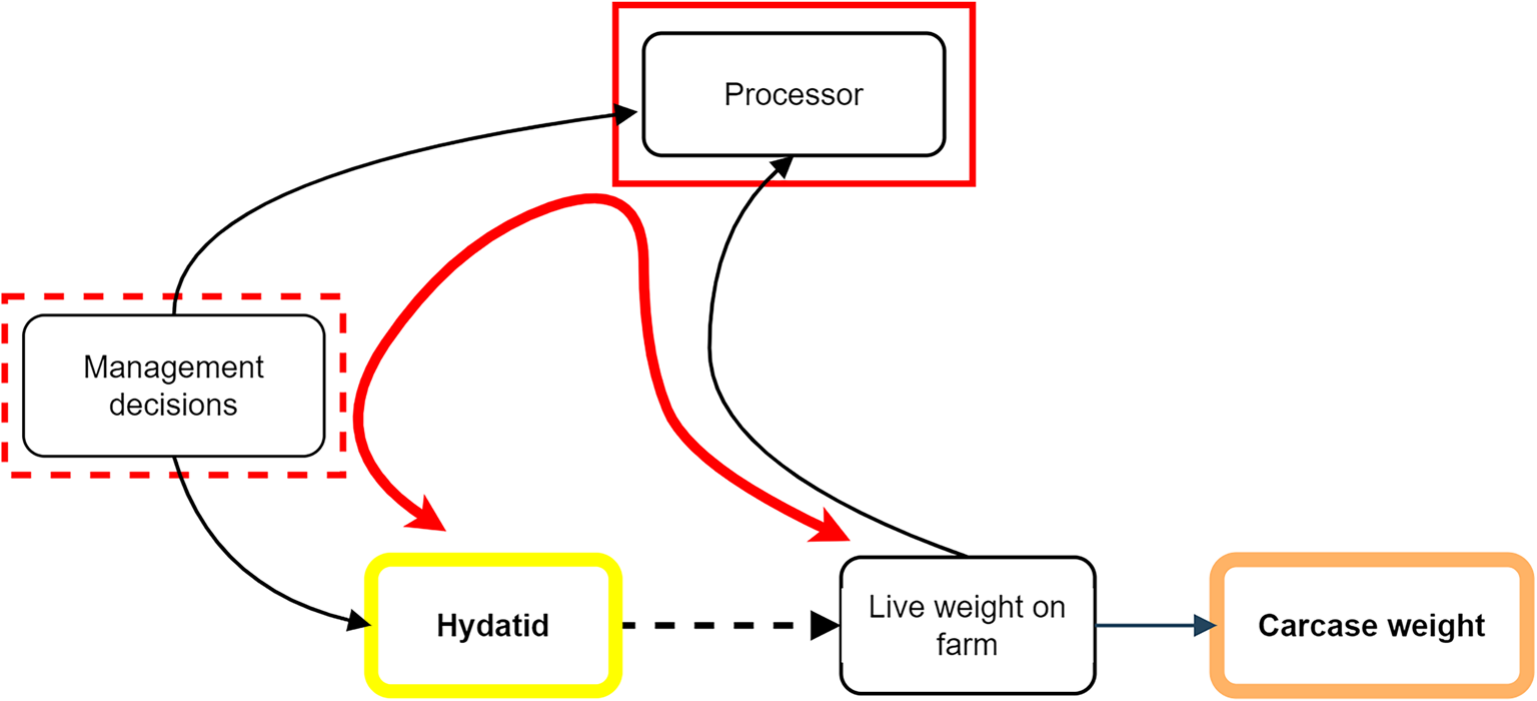
Directed acyclic graph of a mechanism for selection bias of the estimated effect of cystic echinococcosis on carcase weight, via processor. Management decisions influence cystic echinococcosis and a producer’s choice of processor, but live weight subsequently achieved on farm can also influence where an animal is eventually processed. The variable, ‘Processor’, is inherently controlled (red box), because the dataset is from selected processors, and is also controlled within the analysis. The variable ‘management decisions’ is only partially controlled by PIC region (an ancestor variable), and whether cattle are grainfed, and their sex and age (dentition).

The selection of processors in the study could induce selection bias via a ‘Berkson bias’ mechanism that is also worth considering. For example, if processors are more likely to have attracted producers from regions which are systematically more (or less) likely to have cattle with hydatid cysts (producers in northern PIC regions generally go to Queensland processors, and those in southern PIC regions where cystic echinococcosis is less prevalent generally go to processors in southern states), and these processors also target cattle of particular weights for specific markets, an apparent statistical association would be created between cystic echinococcosis and carcase weight (selection bias; Fig. 12). Overall, we believe selection bias via this mechanism is unlikely due to the diverse geographic range of the processors in this study and their large source regions. This pathway of selection bias (Fig. 12) was also partially blocked by variables associated with ‘management decision’ that were included to control confounding.

Lastly, measurement error and misclassification of any variables included in the analyses could cause information bias of the effect of cystic echinococcosis on carcase weight. The sensitivity and specificity of hydatid cyst detection at processing can be low^41^ (although in this study, is likely to be improved due to recording of comorbidities) and is likely to vary between meat inspectors and processors, and with numbers, sizes and locations of cysts in cattle. As mentioned above, it has been suggested that if cysts do affect weight, then many and large cysts could be more likely to have a greater effect on weight^13,38^. The number and size of cysts was not reported in the dataset used in the current study; however, in a previous study in Australia, we found that the probability of detection was higher in cattle with multiple cysts (data not available on detection probability according to size or location of cysts)^41^. Therefore, cattle with a greater number of cysts are more likely to be represented in this dataset; consequently, we can expect that a true effect would not be greater than our estimated effect. Regarding detection differences between sites, the liver and lungs are the main predilection sites for hydatid cysts in intermediate hosts^5,42,43^, and most studies of Australian cattle report cysts either solely in the liver or in both liver and lungs (less commonly lung only)^1,4,44^. This is consistent with findings in the current study. Unusually, one study of Australian cattle arriving in Japan found cysts predominantly in the lungs^45^. Another from Chile found that cysts were more likely to be solely in the lungs in young cattle or when cattle were co-infected with fluke^46^. Unlike the current study, findings in all these previous studies were not consistently stratified by major confounding factors such as age, comorbidities including fluke, and origin of cattle; therefore, differences in proportions of cysts in various locations are difficult to interpret and the findings in the current study should not be interpreted as misclassification. In addition, in the current study, the geographic distributions of cystic echinococcosis and fluke in Australia are consistent with known epidemiology (wild dogs in the Great Dividing Range are considered important hosts for *Echinococcus granulosus*, and coastal, warmer regions are areas in which water and the snail involved in the lifecycle of *Fasciola hepatica* [fluke] is found) and demonstrated relatively different geographic distributions^47,48^. Therefore, whilst some cattle with cystic echinococcosis in the lungs but not the liver (if fluke co-infection truly influenced cyst location) might have been misclassified as negative (if lung cysts are less likely to be detected), the proportion of cattle in which this would have occurred would have been small in this Australian context and we consider it would have little influence on the effect estimate in the current study. Regarding misclassification bias associated with processors, in the current study, this potential source of information bias was accounted for by including processors in the analyses; however, this will not account for differences within processors (variation between inspectors) or regional differences. Whilst the variation between inspectors within each processing site could be considered relatively low (they are all trained on the same pathways and learn from each other at the same site), regional differences could be marked due to the spatial variation in relative frequency of other diseases. For example, hydatid cysts might be more readily detected in cattle from northern PIC regions because fluke (another parasitic condition found in the liver) is less likely in this region and does not provide a competing diagnosis. If there is a higher probability of hydatid detection in northern PIC regions where cattle are inherently lighter, this effect could be incorrectly attributed to hydatid cysts rather than changes in detection probability, accounting for the effect estimated in this study. It is known that increasing time for meat inspection increases the sensitivity of disease detection^49^, and although processors in which multiple morbidities can be recorded were selected for this study, it is likely that recording is still influenced by processing line speed and that the most obvious or expected conditions would be recorded first, followed by other conditions as time allows. The influence of bias due to differing probability of hydatid detection in PIC regions in this study is minimised by the inclusion of PIC region as a covariate in the statistical analyses.

As well as the sources of bias discussed above, another limitation of the data used in this study is recording of prevalent, not incident, exposures and outcomes. Estimation of an unbiased effect measure using prevalence data depends on an underlying stable cohort so that exposure histories are comparable between groups, with known incidence and duration of cystic echinococcosis. Whilst the analysis was stratified by age and year of processing to increase stability within groups, dentition was used as a proxy for age in this study, which is a broad representation of actual age. To determine subtle effects (for example, a producer keeping cattle for a few extra weeks to reach a target weight because they were slower to gain weight due to cystic echinococcosis) would require knowledge of days since infection with hydatid to determine if weight gain is slowed by infection. Even with accurate age data, cattle will be exposed at different times depending on their herd circumstances (therefore, duration of time with hydatid will vary). The effect becomes more marked as cattle reach eight-teeth because they can be any age > 3.5 years. Cattle that are 3.5 years old are more likely to be in better body condition than those that are 10 years old. Older cattle are also more likely to have detectable hydatid cysts due to the longer exposure time to *Echinococcus* eggs in the environment and the longer time for cysts to develop in their viscera^38^.

## Conclusion

The small reduction in carcase weight due to the presence of hydatid cysts that was estimated in this study is a biologically plausible effect. Major sources of bias such as the influence of sex, age, comorbidities (including fluke) could be accounted for; therefore, this estimate is vastly reduced from previous crude estimates^10,11,14,15^. However, some sources of confounding, selection and information bias could not be ruled out and would most likely bias the estimate towards a negative effect on carcase weight as observed in our analyses; overall, it is likely that cystic echinococcosis has no appreciable effect on carcase weight in cattle.

Ultimately, if valid, such a small reduction in carcase weight would be a very small percentage of liveweight for most cattle and difficult to observe. Whilst this weight difference might be valuable at population level, it is debatable whether it would be sufficient for producers to be motivated to undertake greater control measures against hydatid infection in their cattle. A cost–benefit analysis (CBA) would need to be conducted to determine the value of development and implementation of control strategies (for example, vaccination) to producers and processors.

We believe that further collection of observational prevalence-based data is unlikely to further refine our estimate of the effect of cystic echinococcosis on carcase weight, given the difficulty of measuring age of cattle and time onset of disease, as well as potential common causes of cystic echinococcosis and carcase weight. Given the current findings, we suggest that if a CBA indicates an economically viable impact of control measures, a field-trial in which control strategies are randomised to cattle would need to be conducted. However, it should be noted that the study would be logistically difficult because a large number of cattle would be required to detect small differences in weight, and the cattle would need to be followed to processing with accurate records from birth.

A finding in the study that should not be overlooked is the high prevalence of cystic echinococcosis in cattle in the Brisbane region. This has not previously been highlighted. To reduce the risk of echinococcosis in people, cattle producers and domestic dog owners in this region should be targeted by public health interventions to ensure safe disposal of cattle and other intermediate hosts (macropods and sheep) that die on farm and the use of effective anthelmintics in dogs.

### Supplementary Information

Supplementary Information.

## Data Availability

Supporting data are available from https://github.com/vikijbrookes/hydatid.
