# Supplementary material for: The effect of cystic echinococcosis (hydatid disease) on carcase weight in cattle in eastern Australia
Source: Sci Rep. 2024 Mar 27;14:7297. doi: 10.1038/s41598-024-57886-2 (PMC10973430; doi:10.1038/s41598-024-57886-2)
Supplement: Supplementary file 1 — Supplementary Information. [file 41598_2024_57886_MOESM1_ESM.docx]

**The effect of cystic echinococcosis (hydatid disease) on carcase weight in cattle in eastern Australia**

Victoria J. Brookes^a^, Tamsin S. Barnes^b,c^, David J. Jenkins^d^, Matthew Van der Saag^e^, Robert Dempster^f^, Cara S. Wilson^d,g^

^a^Sydney School of Veterinary Science, Faculty of Science, The University of Sydney, Camperdown, NSW, 2008, Australia

^b^The University of Queensland, School of Veterinary Science, Gatton, QLD, 4343, Australia

^c^Epivet Pty. Ltd., Withcott, QLD, 4352, Australia

^d^School of Agricultural, Environmental and Veterinary Sciences, Faculty of Science and Health, Charles Sturt University, Wagga Wagga, NSW, 2678, Australia

^e^Meat and Livestock Australia, 40 Mount Street, North Sydney, NSW, 2060, Australia

^f^Virbac (Australia) Pty Ltd, 361 Horsley Road, Milperra NSW 2214, Australia

^g^CQUniversity Institute for Future Farming Systems, Rockhampton, QLD, 4702, Australia

Corresponding author: Cara Wilson, Building 361, CQIRP CQUniversity, Ibis Avenue, North Rockhampton, QLD, 4702, Australia. +61 0457 041 19 [c.s.wilson@cqu.edu.au](mailto:c.s.wilson@cqu.edu.au)

**Supplementary Material**

Figures Page 2

Tables Page 13

**Figures**


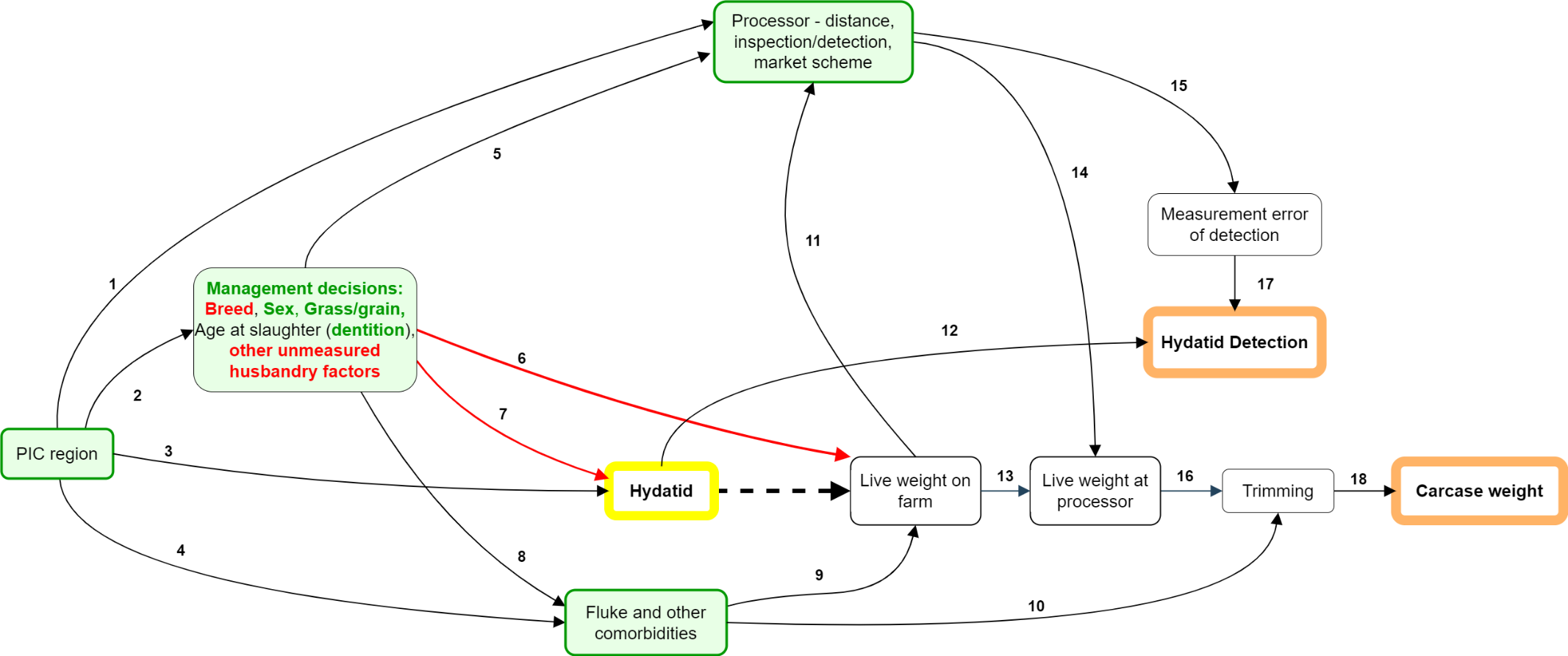


**Figure S1** Directed acyclic graph for the estimation of the total effect of cystic echinococcosis (yellow border) measured as ‘hydatid detection’ (orange border) on carcase weight (orange border - measured as hot standard carcase weight) of cattle. Boxes with green backgrounds indicate variables controlled in analyses (green border = all variables measured and controlled, green text = specific variables measured and controlled, red text unmeasured variables).

Confounding (backdoor) pathways from ‘Hydatid’ to ‘Carcase weight’ are controlled via measured variables ‘Processor (Path 14) and ‘Fluke and other comorbidities’ (Paths 9 and 10). The variable ‘PIC region’ is controlled as a surrogate confounder (Path 2) to eliminate some confounding due to the pathway between ‘Hydatid’ and ‘Live weight’ via ‘Management decisions’ (Paths 6 and 7). Whilst some of the variables in the ‘Management decisions’ group can be controlled (sex, age [dentition], and whether grass- or grain-fed), others are unmeasured, such as breed and proximity to wild dog habitat; however, there is likely a strong association between these unmeasured confounders and PIC region. Dependent measurement and misclassification errors that could cause information bias are also blocked via ‘Processor’ (Path 15). Overall, inclusion of variables in green as covariables in the regression equations isolate the causal effect of Hydatid on Carcase weight, but it is possible that remaining confounding could occur from Hydatid to Live Weight via Paths 6 and 7.


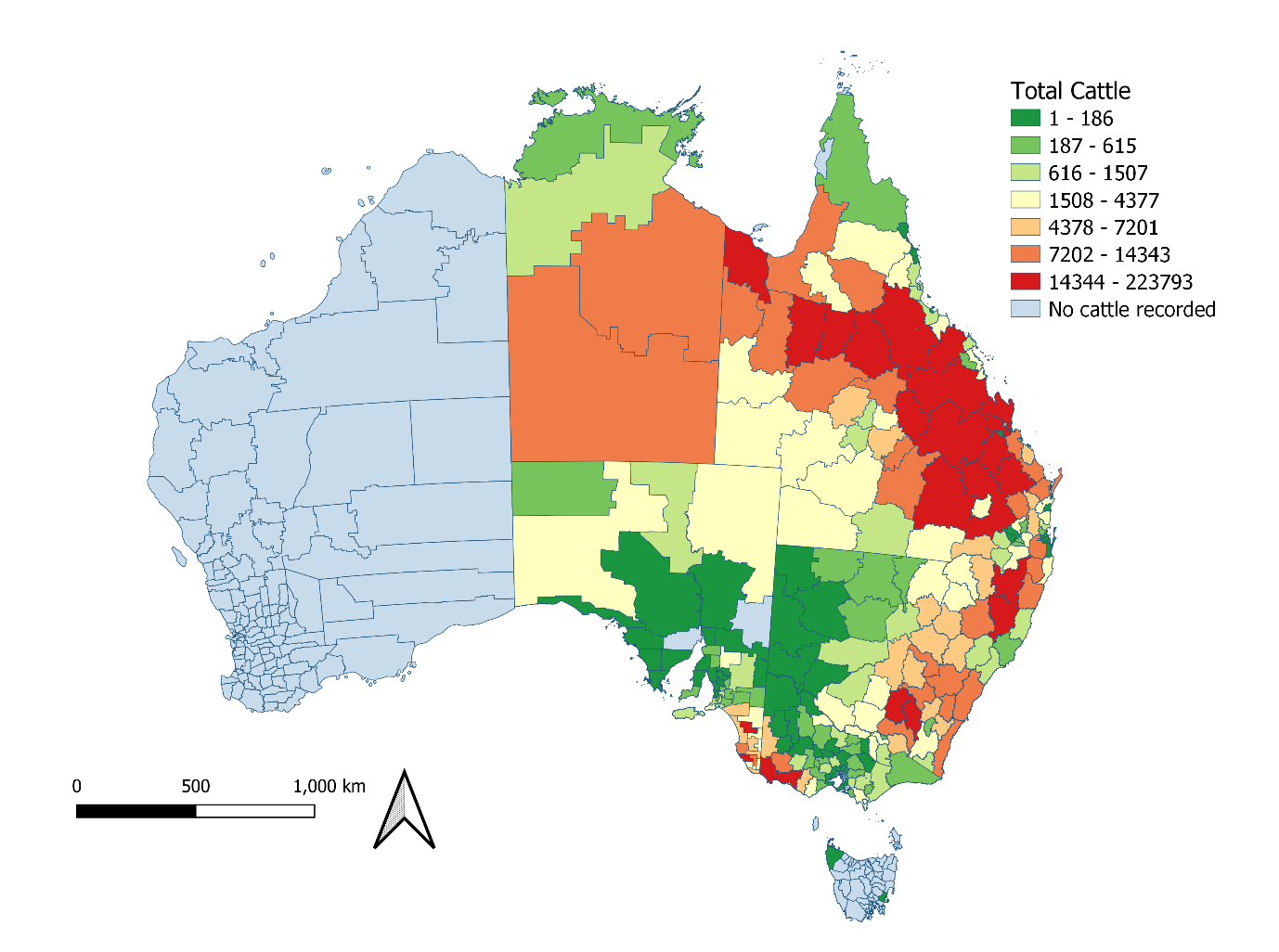


Figure S2 Number of adult cattle with the same property identification code (PIC) region recorded at birth and prior to processing from each PIC region, in a study of the effect of cystic echinococcosis on carcase weight at five processors in eastern Australia, January 2019 – July 2022. Map made by authors using QGIS ^1^.


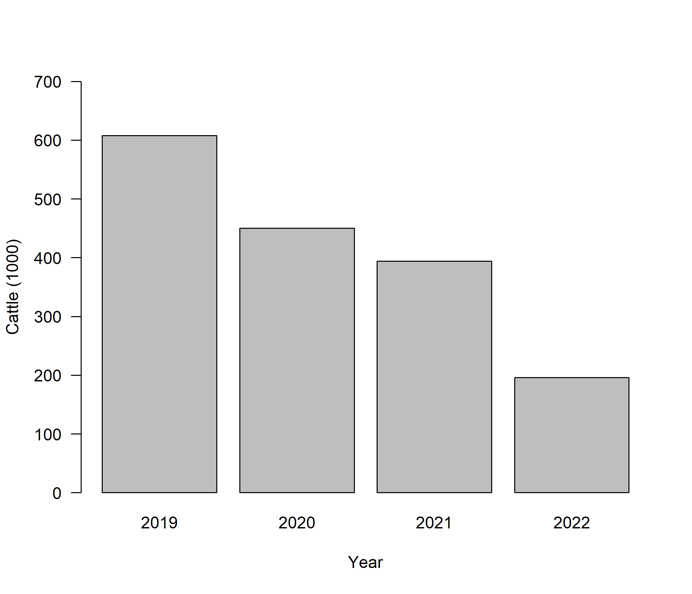


Figure S3 Annual number of adult cattle with the same property identification code (PIC) region recorded at birth and prior to processing, in a study of the effect of cystic echinococcosis on carcase weight at 5 processors in eastern Australia, January 2019 – July 2022.


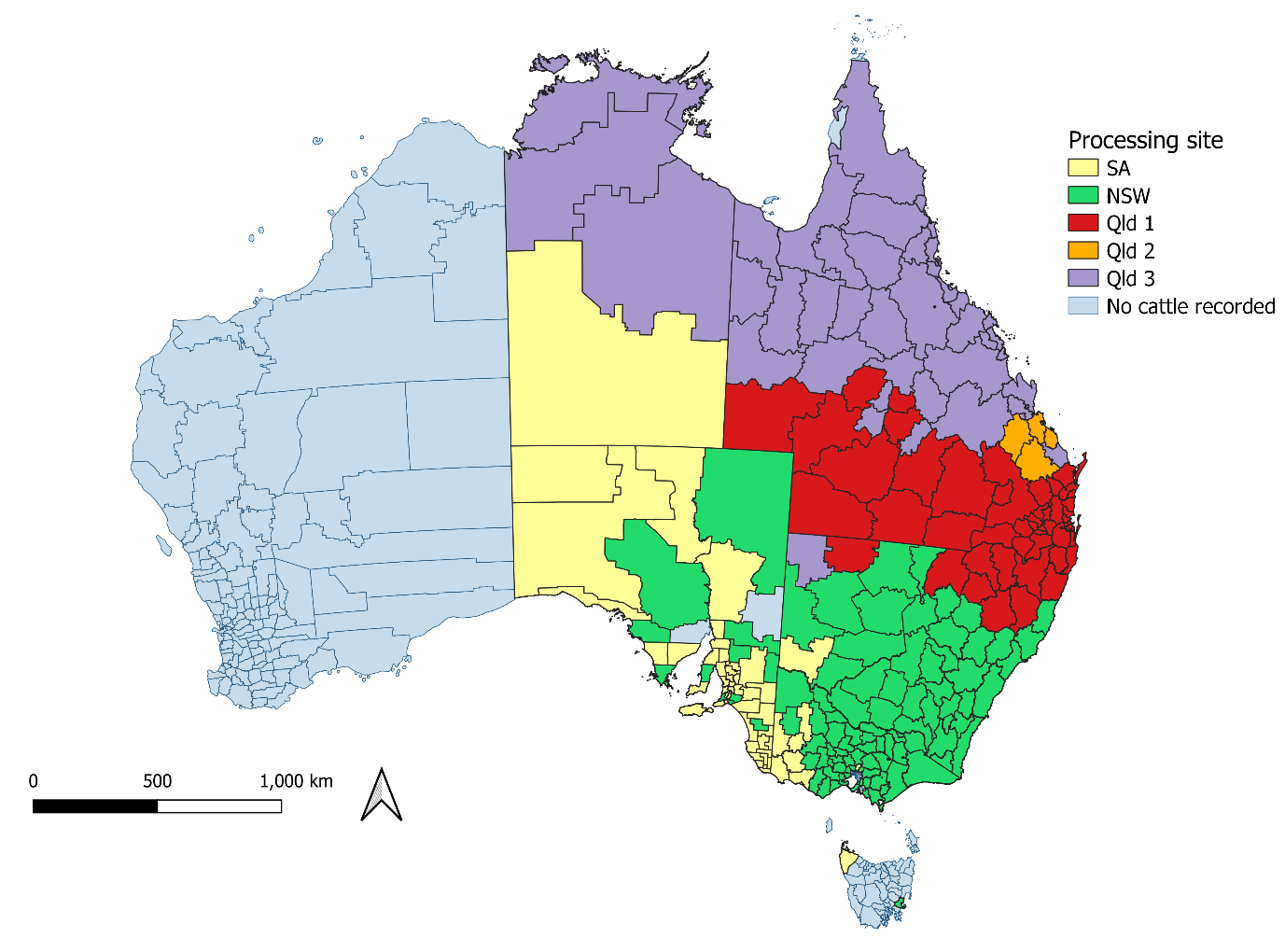


**Figure S4** Most frequent processing site for adult cattle from each property identification code (PIC) region in a study of the effect of cystic echinococcosis on carcase weight at five processors in eastern Australia, January 2019 – July 2022. Cattle had the same PIC region recorded at birth and prior to processing. Map made by authors using QGIS ^1^.


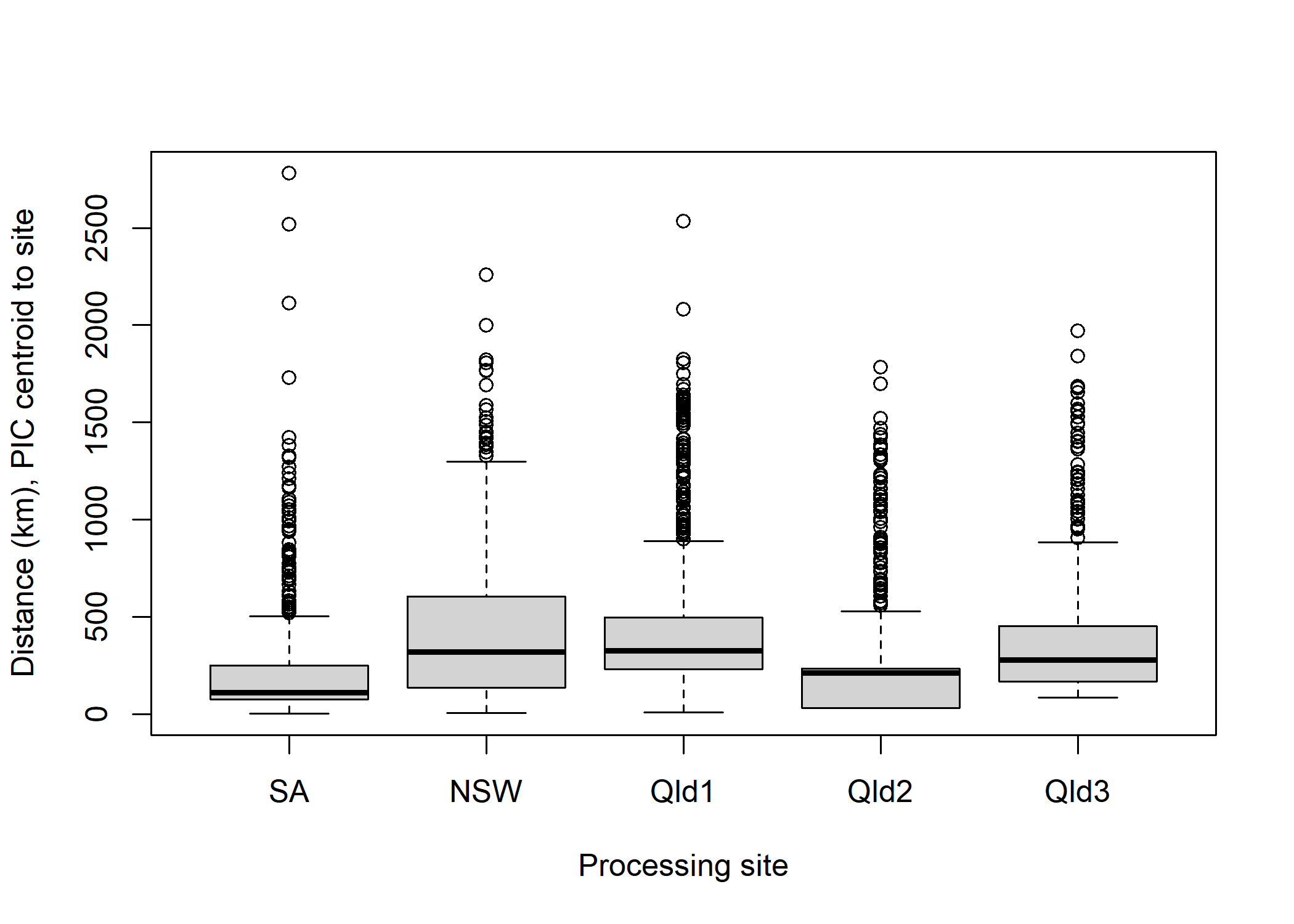


**Figure S5** Geographic range of PIC region centroid to processing site for adult cattle in a study of the effect of cystic echinococcosis on carcase weight at 5 processors in eastern Australia, January 2019 – July 2022. Cattle had the same PIC region recorded at birth and prior to processing.


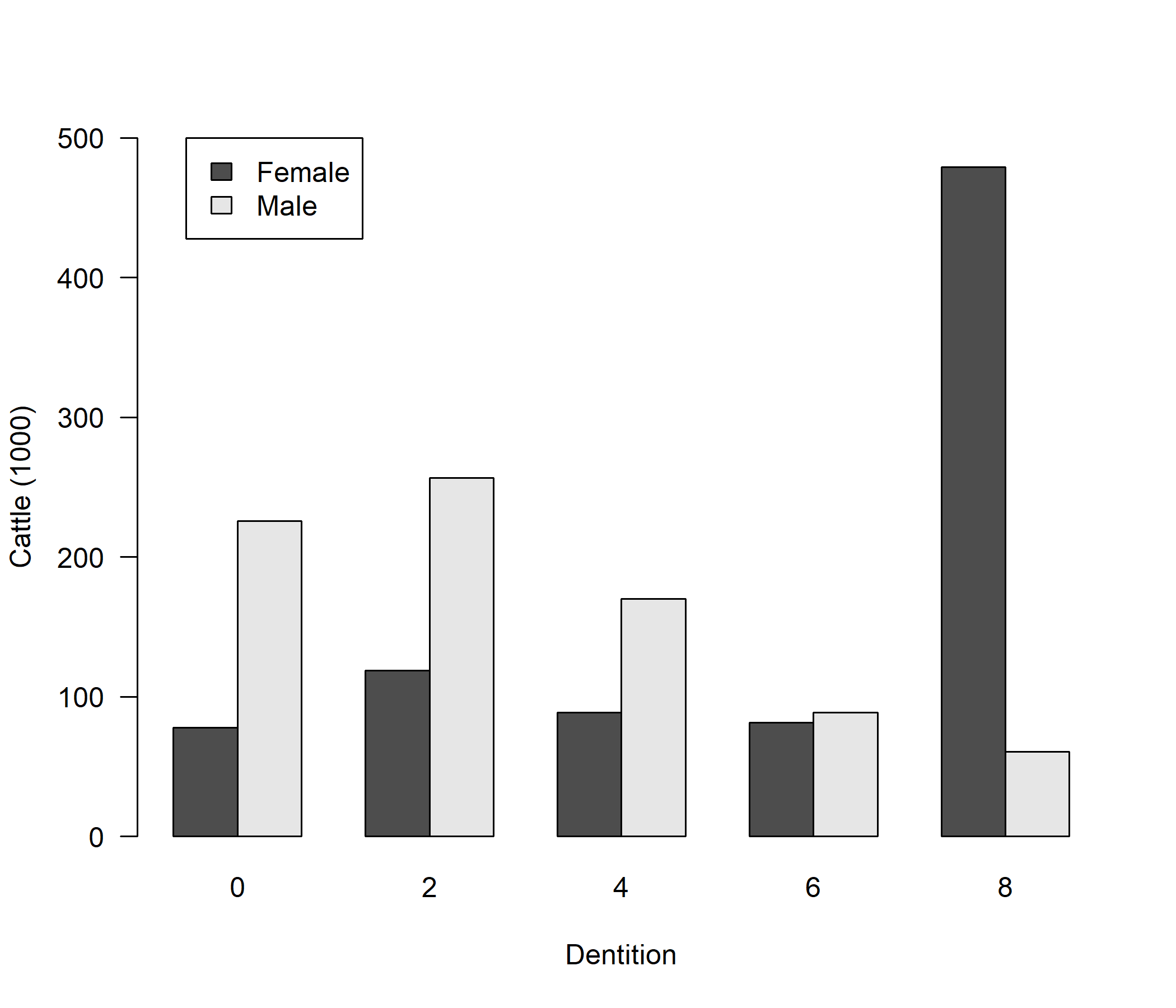


**Figure S6** Number of adult cattle with the same PIC region recorded at birth and prior to processing, by dentition and sex, in a study of the effect of cystic echinococcosis on carcase weight at five processors in eastern Australia, 2019-2022.


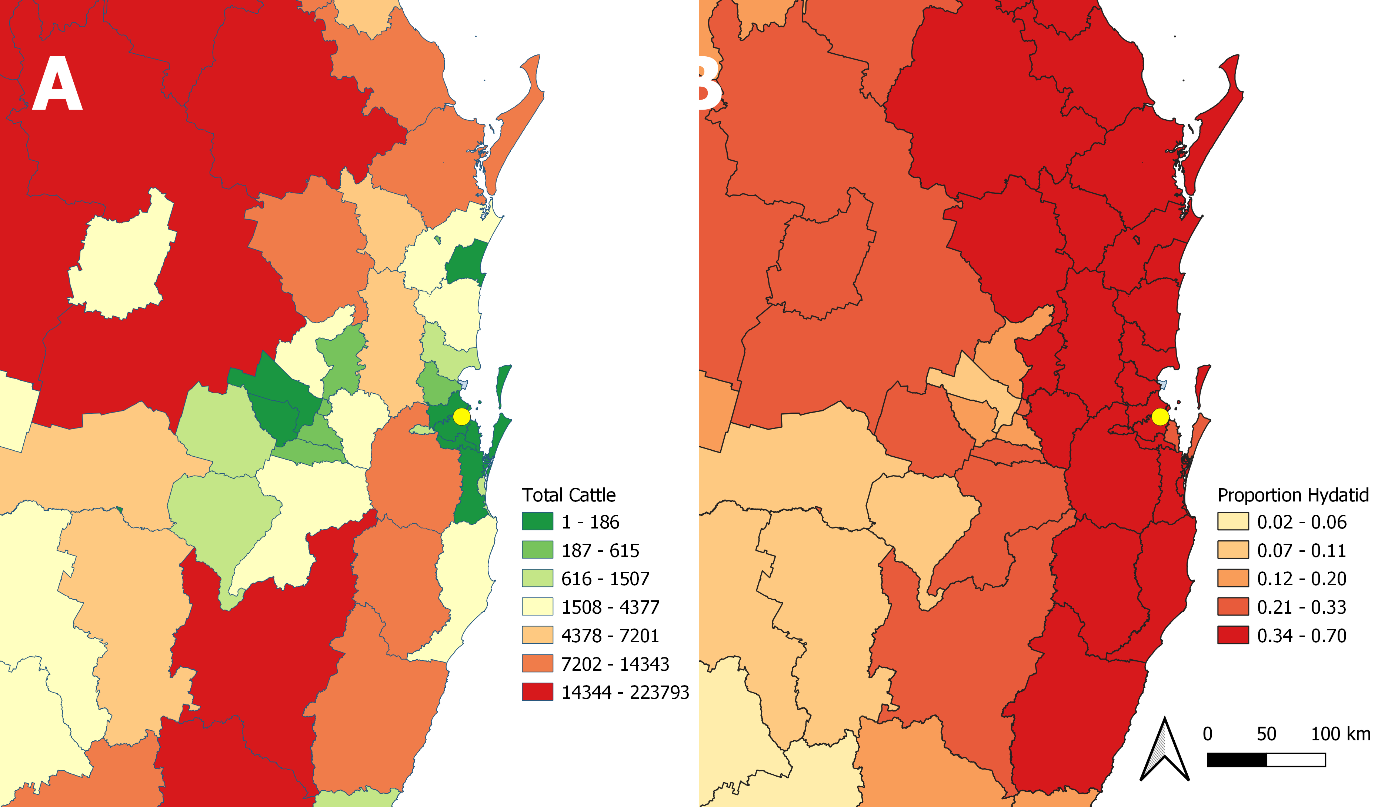


**Figure S7** Choropleth map of the Brisbane region, southern Queensland for adult cattle with the same PIC region recorded at birth and prior to processing, A: number processed, and B: proportion in which hydatid cysts were detected at processing, in a study of the effect of cystic echinococcosis on carcase weight at five processors in eastern Australia, 2019-2022. Map made by authors using QGIS ^1^.


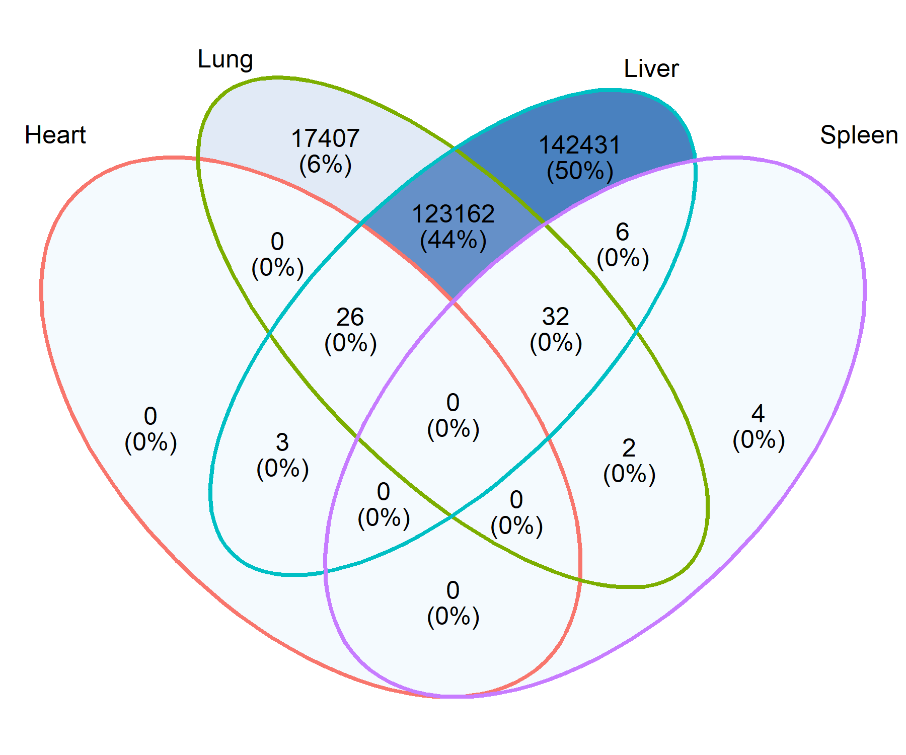


**Figure S8** Venn diagram of the numbers and proportions of adult cattle with the same PIC region recorded at birth and prior to processing, in which hydatid cysts were detected the liver, lungs, spleen and heart, in a study of the effect of cystic echinococcosis on carcase weight at five processors in eastern Australia, 2019-2022.


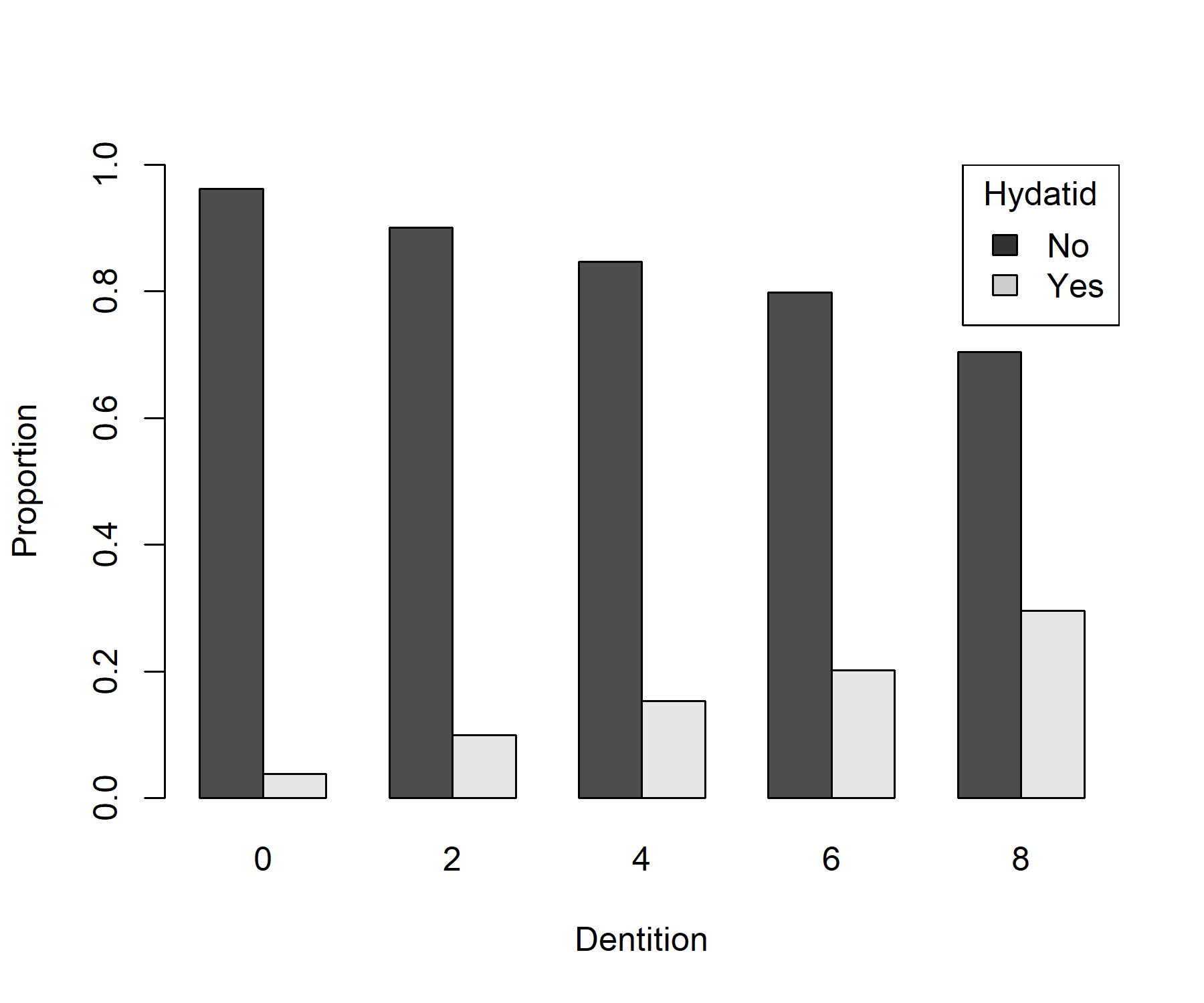


**Figure S9** Barplot of the proportion of adult cattle with the same property identification code (PIC) region recorded at birth and prior to processing in which hydatid cysts were detected in any organ stratified by age, in a study of the effect of cystic echinococcosis on carcase weight at five processors in eastern Australia, 2019-2022.


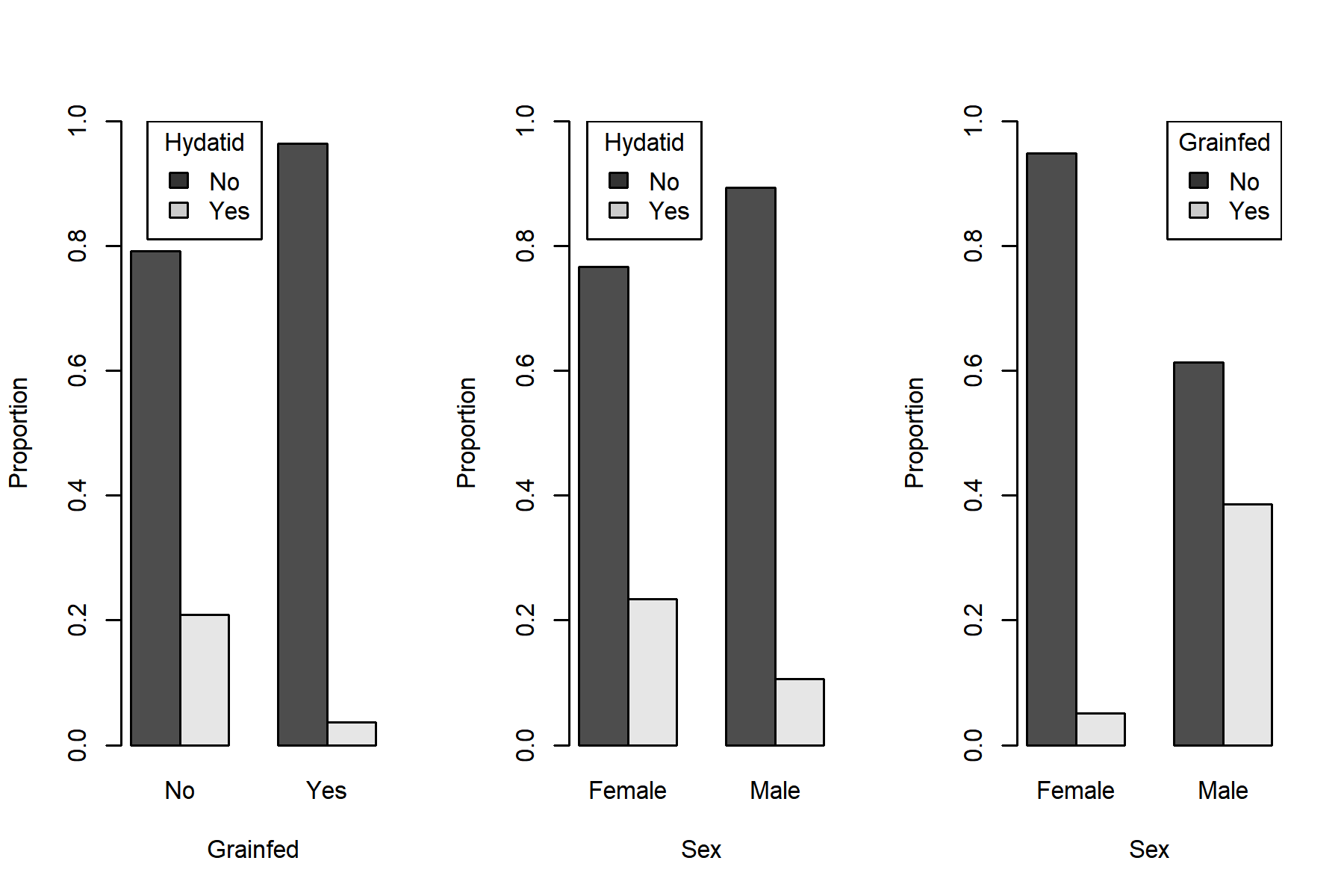


**Figure S10** Barplots of the proportion of adult cattle with the same property identification code (PIC) region recorded at birth and prior to processing, by hydatid cyst detection in any organ, sex, and whether they were grain-fed, in a study of the effect of cystic echinococcosis on carcase weight at five processors in eastern Australia, 2019-2022.


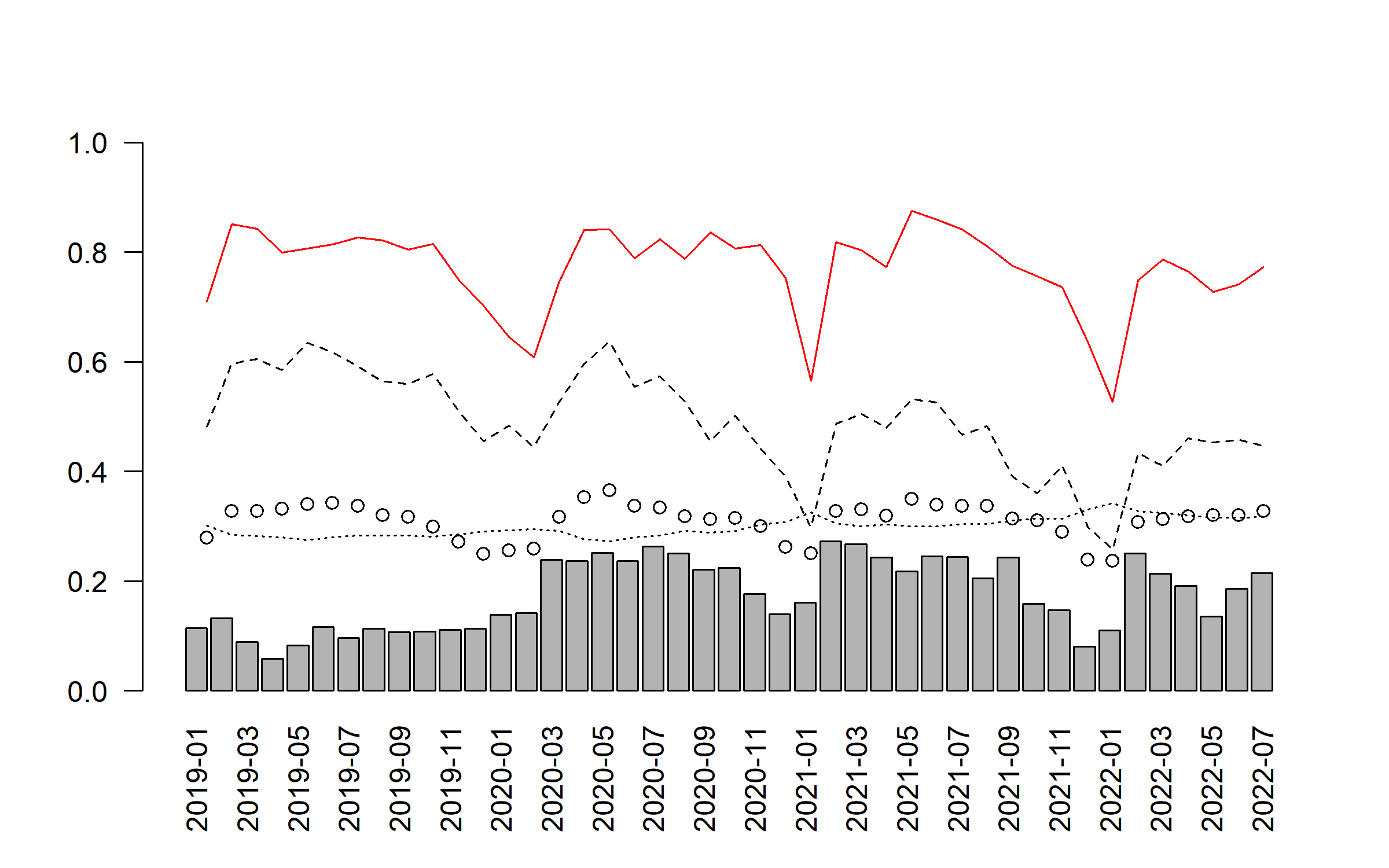


**Figure S11** Timeseries of monthly mean proportion of adult cattle in which hydatid cysts were detected (barplot), were not grain-fed (red line), were female (dashed line), carcase weight (/1000; dotted line), and age by dentition (/10; circles), in a study of the effect of cystic echinococcosis on carcase weight at five processors in eastern Australia, 2019-2022.


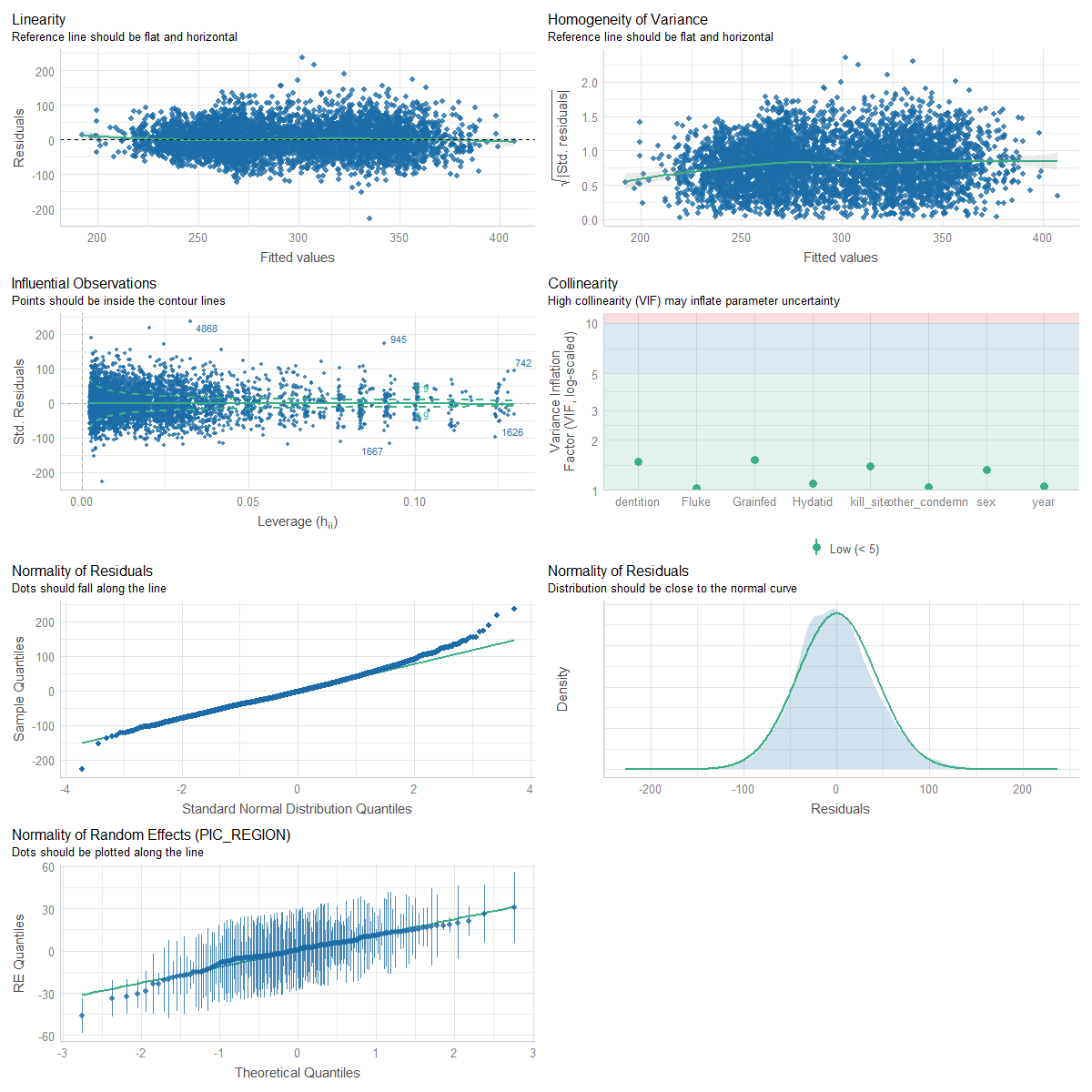


**Figure S12** Plots to assess model assumptions for normality of residuals, normality of random effects, linear relationship, homogeneity of variance, and multicollinearity in a linear mixed-effects regression model of the total effect of hydatid detection on hot standard carcase weight, including year of processing and covariates to reduce confounding as fixed effects (sex, presence of comorbidities, presence of fluke, abattoir, grain-fed or not) and PIC region as a random effect), in a study of the effect of cystic echinococcosis on carcase weight at five processors in eastern Australia, 2019-2022.


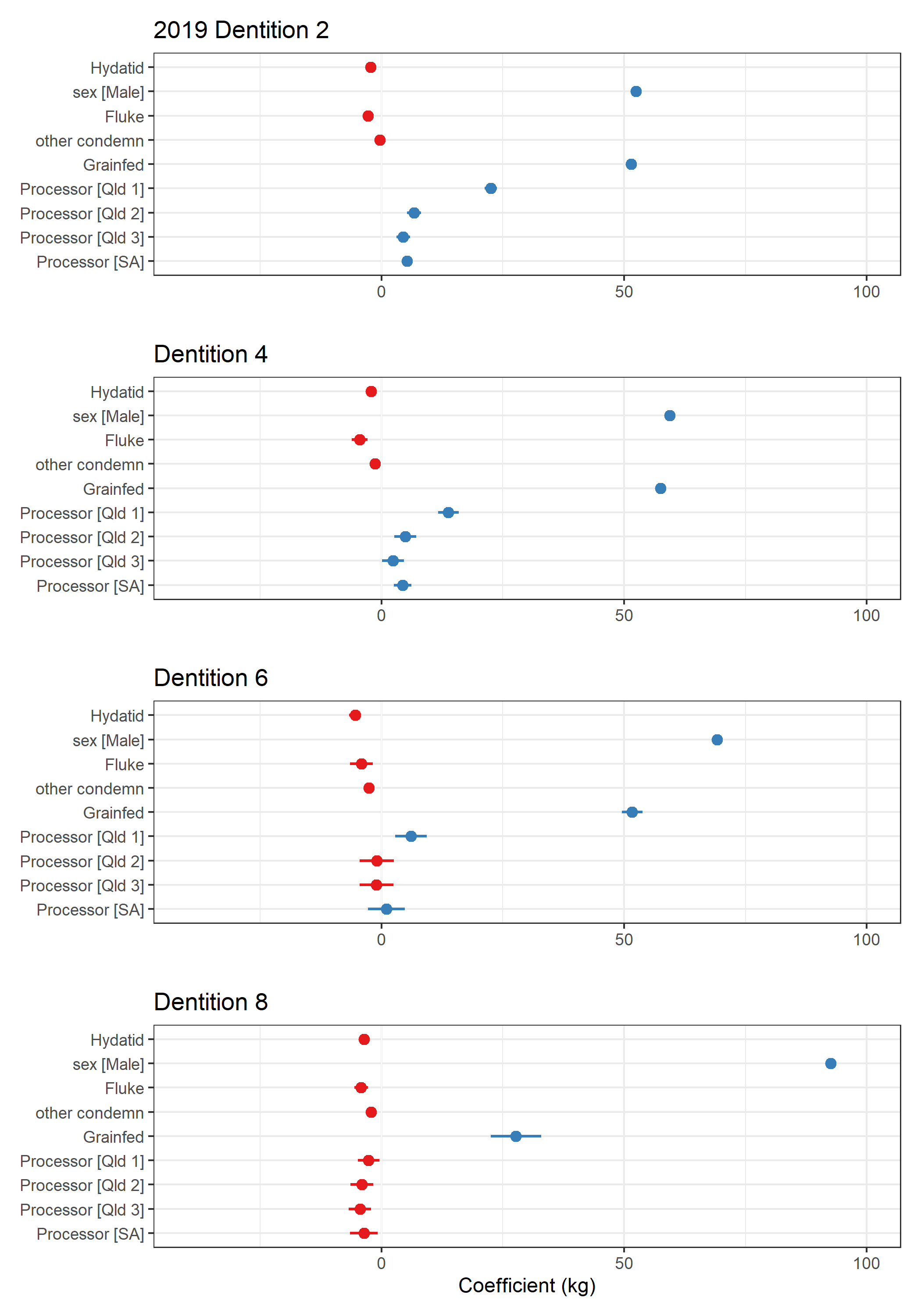


**Figure S13** Plots of fixed effect coefficients (bars = standard error) in linear mixed-effects regression models of the effect of hydatid detection on hot standard carcase weight in cattle processed in 2019, in a study of the effect of cystic echinococcosis on carcase weight at five processors in eastern Australia, 2019-2022.

**
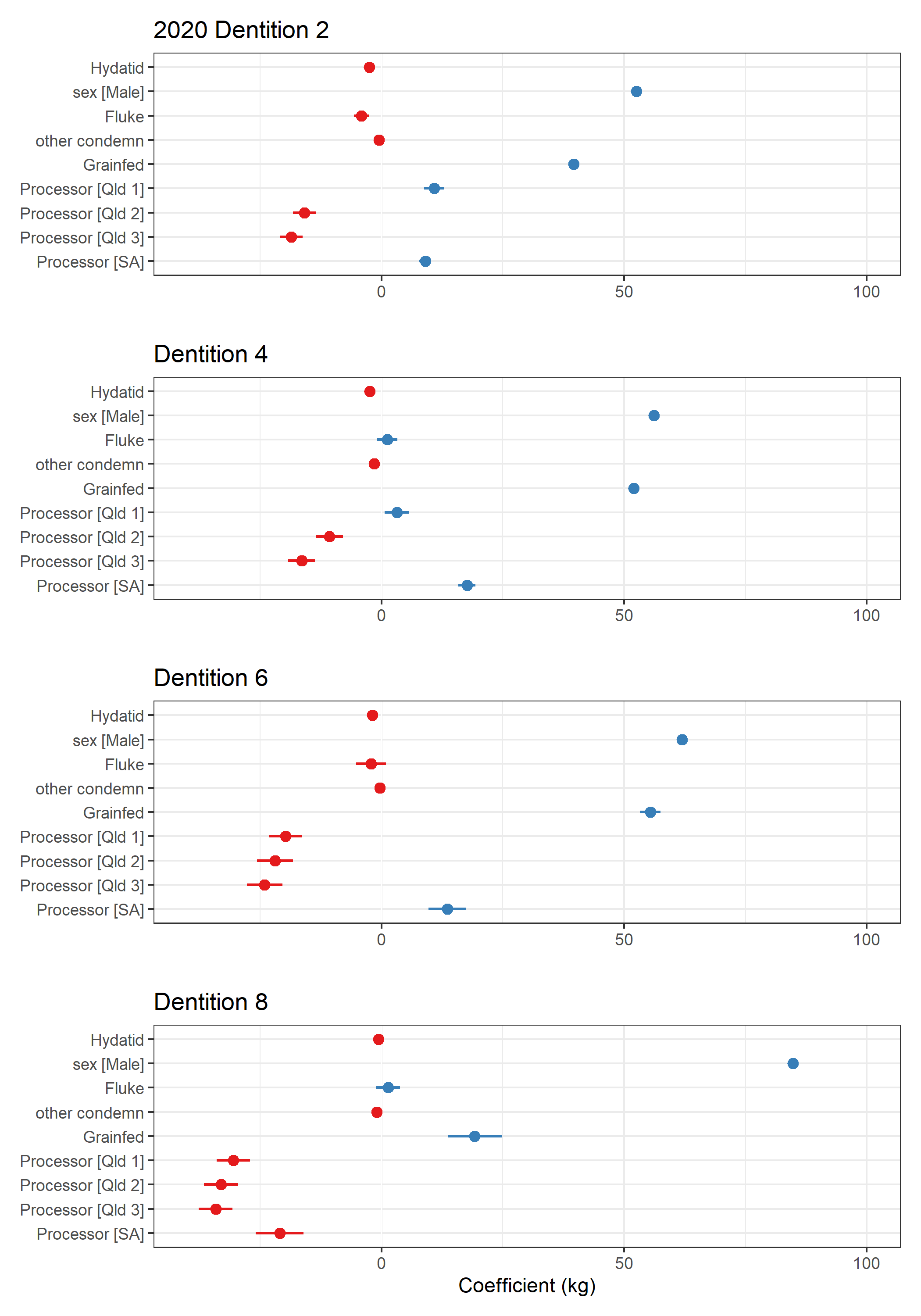
**

**Figure S14** Plots of fixed effect coefficients (bars = standard error) in linear mixed-effects regression models of the effect of hydatid detection on hot standard carcase weight in cattle processed in 2020, in a study of the effect of cystic echinococcosis on carcase weight at five processors in eastern Australia, 2019-2022.


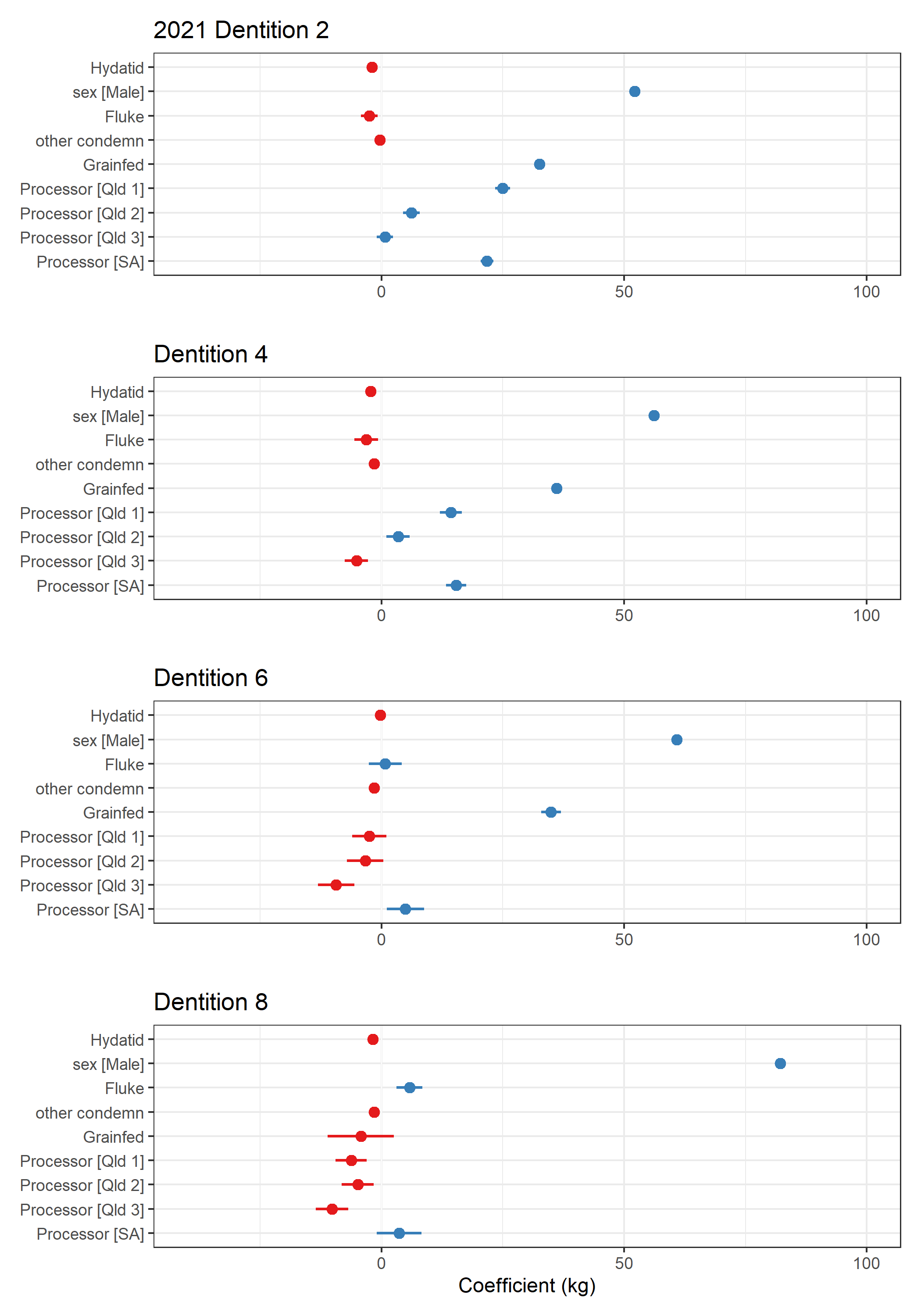


**Figure S15** Plots of fixed effect coefficients (bars = standard error) in linear mixed-effects regression models of the effect of hydatid detection on hot standard carcase weight in cattle processed in 2021, in a study of the effect of cystic echinococcosis on carcase weight at five processors in eastern Australia, 2019-2022.


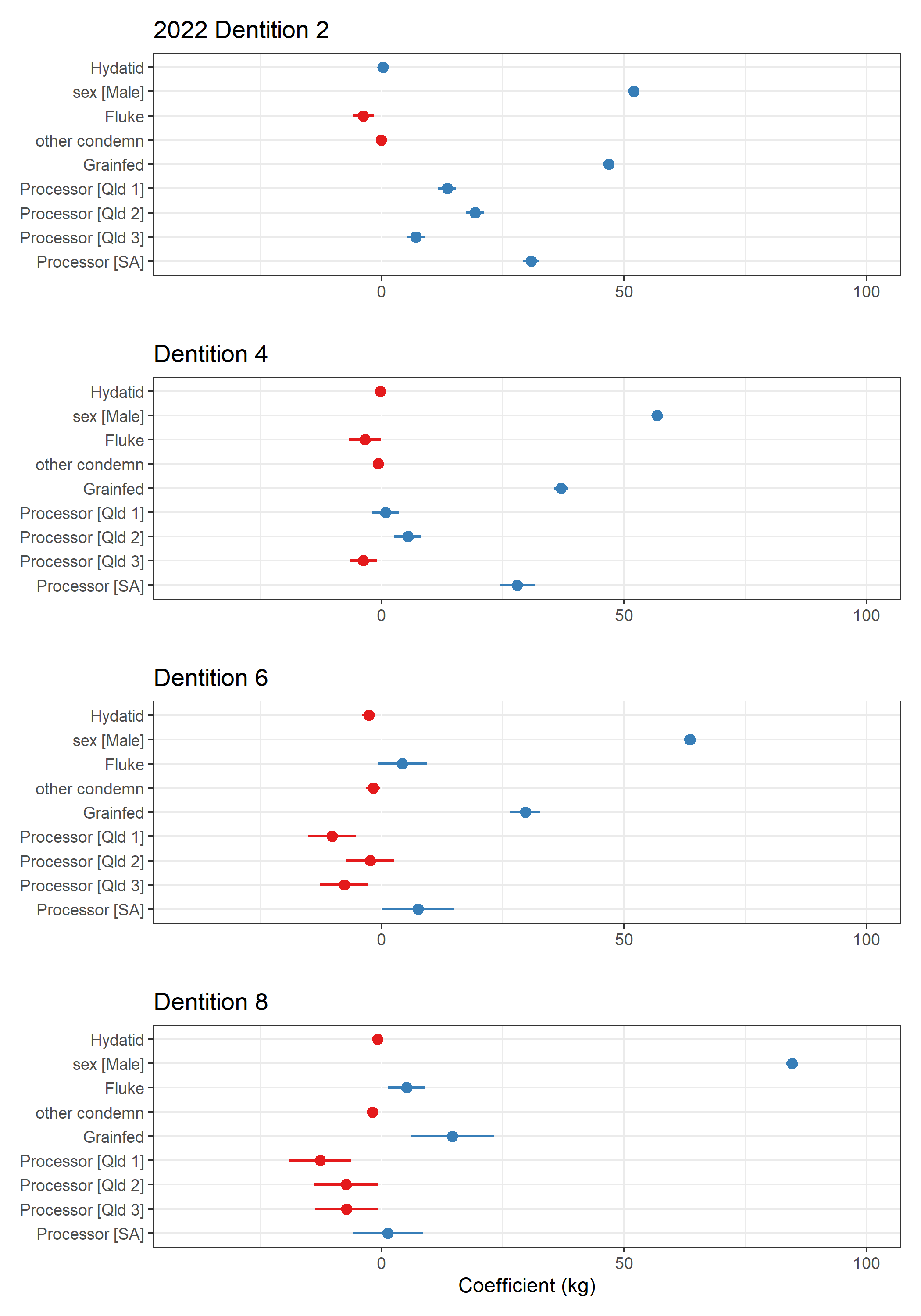


**Figure S16** Plots of fixed effect coefficients (bars = standard error) in linear mixed-effects regression models of the effect of hydatid detection on hot standard carcase weight in cattle processed in 2022, in a study of the effect of cystic echinococcosis on carcase weight at five processors in eastern Australia, 2019-2022.

**Tables**

**Table S1** Estimated total effect of the presence of hydatid cysts (detected at slaughter) on hot standard carcase weight (HSCW; kg) in linear mixed-effects regression analyses of adult cattle which had the same PIC region recorded at birth and prior to processing in 2019, stratified by age (dentition), in a study of cattle processed at five processors in eastern and southern Australia in 2019-2022. HSCW = hot standard carcase weight, se = standard error, σ2 = random effect variance, τ00 = random intercept variance, ICC = intra-class correlation coefficient, N = number of random effect groups, R2 = r-squared value.

| Model | Two-tooth | Four-tooth | Six-tooth | Eight-tooth |
| --- | --- | --- | --- | --- |
| HSCW, kg | Estimate (se) | Estimate (se) | Estimate (se) | Estimate (se) |
| Intercept | 260.00 (1.89) | 259.69 (2.20) | 260.87 (2.48) | 262.79 (2.17) |
| **Hydatid detected** | **-2.28 (0.47)** | **-2.11 (0.52)** | **-5.45 (0.63)** | **-3.59 (0.29)** |
| Covariates: sex, presence of comorbidities, presence of fluke, abattoir, grain-fed (yes/no) | | | | |
|  | | | | |
| **Random Effects (PIC region)** | | | | |
| σ2 | 1511.37 | 1599.42 | 1846.57 | 1865.30 |
| τ00 | 595.30 | 747.31 | 878.20 | 729.29 |
| ICC | 0.28 | 0.32 | 0.32 | 0.28 |
| N | 595.30 | 747.31 | 878.20 | 729.29 |
|  |  |  |  |  |
| Observations | 136104 | 82465 | 54716 | 210412 |
| Marginal R2 / Conditional R2 | 0.437 / 0.596 | 0.406 / 0.595 | 0.340 / 0.552 | 0.196 / 0.422 |

**Table S2** Estimated total effect of the presence of hydatid cysts (detected at slaughter) on hot standard carcase weight (HSCW; kg) in linear mixed-effects regression analyses of adult cattle which had the same PIC region recorded at birth and prior to processing in 2020, stratified by age (dentition), in a study of cattle processed at five processors in eastern and southern Australia in 2019-2022. HSCW = hot standard carcase weight, se = standard error, σ2 = random effect variance, τ00 = random intercept variance, ICC = intra-class correlation coefficient, N = number of random effect groups, R2 = r-squared value.

| **Model** | **Two-tooth** | **Four-tooth** | **Six-tooth** | **Eight-tooth** |
| --- | --- | --- | --- | --- |
| HSCW, kg | Estimate (se) | Estimate (se) | Estimate (se) | Estimate (se) |
| Intercept | 262.29 (1.75) | 259.38 (1.82) | 253.29 (2.08) | 264.40 (2.16) |
| **Hydatid detected** | **-2.52 (0.41)** | **-2.41 (0.42)** | **-1.92 (0.50)** | **-0.59 (0.25)** |
| Covariates: sex, presence of comorbidities, presence of fluke, abattoir, grain-fed (yes/no) | | | | |
|  | | | | |
| **Random Effects (PIC region)** | | | | |
| σ2 | 1469.73 | 1465.94 | 1648.52 | 1782.02 |
| τ00 | 466.27 | 472.39 | 576.89 | 699.76 |
| ICC | 0.24 | 0.24 | 0.26 | 0.28 |
| N | 195 | 190 | 185 | 178 |
|  | | | | |
| Observations | 100291 | 72918 | 44686 | 148695 |
| Marginal R2 / Conditional R2 | 0.464 / 0.593 | 0.475 / 0.603 | 0.381 / 0.542 | 0.229 / 0.447 |

**Table S3** Estimated total effect of the presence of hydatid cysts (detected at slaughter) on hot standard carcase weight (HSCW; kg) in linear mixed-effects regression analyses of adult cattle which had the same PIC region recorded at birth and prior to processing in 2021, stratified by age (dentition), in a study of cattle processed at five processors in eastern and southern Australia in 2019-2022. HSCW = hot standard carcase weight, se = standard error, σ2 = random effect variance, τ00 = random intercept variance, ICC = intra-class correlation coefficient, N = number of random effect groups, R2 = r-squared value.

| **Model** | **Two-tooth** | **Four-tooth** | **Six-tooth** | **Eight-tooth** |
| --- | --- | --- | --- | --- |
| HSCW, kg | Estimate (se) | Estimate (se) | Estimate (se) | Estimate (se) |
| Intercept | 280.81 (1.93) | 280.39 (2.05) | 277.90 (2.35) | 285.25 (2.66) |
| **Hydatid** | **-1.96 (0.41)** | **-2.25 (0.39)** | **-0.28 (0.49)** | **-1.78 (0.31)** |
| Covariates: sex, presence of comorbidities, presence of fluke, abattoir, grain-fed (yes/no) | | | | |
|  | | | | |
| **Random Effects (PIC region)** | | | | |
| σ2 | 1448.18 | 1464.85 | 1687.08 | 1968.21 |
| τ00 | 604.56 _PIC_REGION_ | 620.29 _PIC_REGION_ | 728.40 _PIC_REGION_ | 1076.83 _PIC_REGION_ |
| ICC | 0.29 | 0.30 | 0.30 | 0.35 |
| N | 195 _PIC_REGION_ | 194 _PIC_REGION_ | 185 _PIC_REGION_ | 182 _PIC_REGION_ |
|  | | | | |
| Observations | 90536 | 72722 | 47555 | 121309 |
| Marginal R2 / Conditional R2 | 0.341 / 0.535 | 0.332 / 0.531 | 0.300 / 0.511 | 0.239 / 0.508 |

**Table S4** Estimated total effect of the presence of hydatid cysts (detected at slaughter) on hot standard carcase weight (HSCW; kg) in linear mixed-effects regression analyses of adult cattle which had the same PIC region recorded at birth and prior to processing in 2022, stratified by age (dentition), in a study of cattle processed at five processors in eastern and southern Australia in 2019-2022. HSCW = hot standard carcase weight, se = standard error, σ2 = random effect variance, τ00 = random intercept variance, ICC = intra-class correlation coefficient, N = number of random effect groups, R2 = r-squared value.

|  | **Two-tooth** | **Four-tooth** | **Six-tooth** | **Eight-tooth** |
| --- | --- | --- | --- | --- |
| HSCW, kg | Estimate (se) | Estimate (se) | Estimate (se) | Estimate (se) |
| Intercept | 274.21 (1.99) | 277.58 (2.35) | 280.50 (2.83) | 290.31 (3.03) |
| **Hydatid** | **0.32 (0.58)** | **-0.26 (0.61)** | **-2.64 (0.69)** | **-0.82 (0.46)** |
| Covariates: sex, presence of comorbidities, presence of fluke, abattoir, grain-fed (yes/no), distance to processor. | | | | |
|  | | | | |
| **Random Effects (PIC region)** | | | | |
| σ2 | 1406.30 | 1508.82 | 1667.65 | 1974.90 |
| τ00 | 538.46 | 656.30 | 843.99 | 1202.25 |
| ICC | 0.28 | 0.30 | 0.34 | 0.38 |
| N | 180 | 168 | 160 | 167 |
|  | | | | |
| Observations | 48806 | 30892 | 23180 | 59352 |
| Marginal R2 / Conditional R2 | 0.393 / 0.561 | 0.328 / 0.531 | 0.296 / 0.533 | 0.230 / 0.521 |

References

1 QGIS Development Team. QGIS Geographic Information System 3.34.1. [*https://www.qgis.org/*](https://www.qgis.org/) (2023).
